# Low Molecular Weight Inhibitors Targeting the RNA-Binding Protein HuR

**DOI:** 10.3390/ijms241713127

**Published:** 2023-08-23

**Authors:** Benjamin Philipp Joseph, Verena Weber, Lisa Knüpfer, Alejandro Giorgetti, Mercedes Alfonso-Prieto, Sybille Krauß, Paolo Carloni, Giulia Rossetti

**Affiliations:** 1 Institute for Neuroscience and Medicine and Institute for Advanced Simulations (INM-9/IAS-5), Computational Biomedicine, Forschungszentrum Jülich, 52425 Jülich, Germany; b.joseph@fz-juelich.de (B.P.J.); v.weber@fz-juelich.de (V.W.); alejandro.giorgetti@univr.it (A.G.); m.alfonso-prieto@fz-juelich.de (M.A.-P.); g.rossetti@fz-juelich.de (G.R.); 2 Faculty of Mathematics, Computer Science and Natural Sciences, RWTH Aachen University, 52062 Aachen, Germany; 3 Institute of Biology, University of Siegen, 57076 Siegen, Germany; lisa.knuepfer@uni-siegen.de; 4 Department of Biotechnology, University of Verona, 37134 Verona, Italy; 5 Jülich Supercomputing Centre (JSC), Forschungszentrum Jülich, 52425 Jülich, Germany; 6 Department of Neurology, RWTH Aachen University, 44517 Aachen, Germany

**Keywords:** RNA-binding protein, human antigen R (HuR), high-throughput virtual screening, small molecule inhibitors, RNA pulldown assay

## Abstract

The RNA-binding protein human antigen R (HuR) regulates stability, translation, and nucleus-to-cytoplasm shuttling of its target mRNAs. This protein has been progressively recognized as a relevant therapeutic target for several pathologies, like cancer, neurodegeneration, as well as inflammation. Inhibitors of mRNA binding to HuR might thus be beneficial against a variety of diseases. Here, we present the rational identification of structurally novel HuR inhibitors. In particular, by combining chemoinformatic approaches, high-throughput virtual screening, and RNA–protein pulldown assays, we demonstrate that the 4-(2-(2,4,6-trioxotetrahydropyrimidin-5(2H)-ylidene)hydrazineyl)benzoate ligand exhibits a dose-dependent HuR inhibition effect in binding experiments. Importantly, the chemical scaffold is new with respect to the currently known HuR inhibitors, opening up a new avenue for the design of pharmaceutical agents targeting this important protein.

## 1. Introduction

Human antigen R (HuR), the protein product of embryonic lethal and abnormal vision gene ELAVL1, binds to and stabilizes messenger RNA (mRNA) transcripts, enhancing their translation into proteins [1,2]. Therefore, it plays a critical role in post-transcriptional gene regulation [3], leading to a variety of processes, including cell proliferation [4], differentiation, stress response [5], and nociceptor function [6]. HuR dysregulation has been implicated in numerous diseases, including cancer [7,8,9,10,11], neurological disorders [12,13,14,15,16], and inflammatory diseases [17,18,19]. This highlights HuR as a very important target for pharmaceutical intervention.

Indeed, several therapeutic strategies have been put forward [20,21,22], from RNA interference [23,24] and immunotherapy [25], to natural compounds [26,27] and small molecule inhibitors [27,28,29]. RNA interference and immunotherapy are still in their early stages for HuR, with several limitations [30]. The use of natural compounds can pose challenges, like variable potency and specificity or limited bioavailability [31]. Instead, therapeutic strategies based on small molecules are potentially very promising. Examples of molecules that have been identified in vitro and in cellulo as HuR inhibitors (including a few low weight natural products) are: sulfonyl aromatics [28], coumarins [27], and flavonoids [26] (see Table S1). A few successful small molecules that have been experimentally proven to bind HuR proteins in vivo include embelin [32], okicenone [29], triptolide [33], leptomycin B [34], selinexor [35], KH-3 and derivatives [36], suramin [37], mitoxantrone [38], and DHTS [39] (see Figure S1). One in vivo tested ligand, MS-444 [40], is currently undergoing preclinical studies [41].

**Figure 1:**
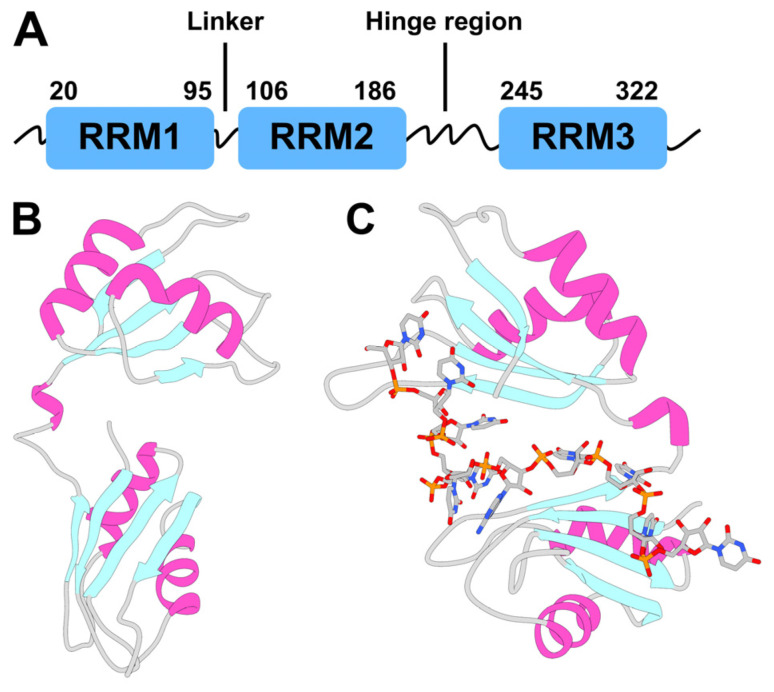
Human antigen R (HuR) protein topology and structure. (**A**) Scheme of the full-length HuR topology. HuR is composed of three highly conserved canonical RNA recognition motifs (RRMs). The residue number shown here corresponds to the UniProt ID Q15717. Each RRM domain adopts a βαββαβ topology, also called a canonical αβ sandwich, where a four-stranded, anti-parallel β-sheet is packed against two α-helices. (**B**,**C**) X-ray structures of HuR RRM1/2 alone (PDB code: 4EGL [45]) and in complex with a 5′-AUUUUUAUUUU-3′ target RNA (PDB code: 4ED5 [45]). RRM2 and RRM1 correspond to the top and bottom domains, respectively.

Structural insights on HuR in the free state and in complex with in vivo ligands have been provided through X-ray crystallography, NMR, and fluorescence polarization assays. The HuR protein contains three RNA recognition motifs (RRM1–3) (Figure 1), responsible for binding adenylate and uridylate (AU)-rich elements (AREs) [2,42] in mRNA, through highly conserved ribonucleoprotein (RNP) sequences [43]. RRM1 and RRM2 are separated by a short, 10 amino acid linker, whereas a longer, ~50 residue hinge region connects the third RRM3 motif (see Figure 1). This hinge bears a nucleocytoplasmic shuttling element [44] that may transport HuR from the nucleus to the cytoplasm [44]. RRM1 initiates HuR’s binding to AREs (K_D_ = 488 nM) [45]. Upon this first binding event, the affinity of RRM2 for AREs increases (from K_D_ = 488 nM to K_D_ = 169 nM) [45]. Therefore, RRM1–2 serve as the HuR anchoring point to the mRNA. X-ray structures are available for HuR in the free state and in complex with RNA [45], showing that the interdomain linker connecting RRM1/2 [24] and the charged surface of these two RRMs forms a cleft (of a transient nature [45]) in the RNA-bound state (Figure 1B,C), with the target RNA fully occupying this cleft [45]. Ligands, such as coumarins [27], 15,16-dihydrotanshinone I (DHTS) [39], and KH-3 [36], as well as their derivatives, also bind to the same transient cleft as RNA; however, these small molecules can usually only partially fill the cleft [23,24]. This impairs the anchoring of RRM1–2 to the mRNA AREs, thus hampering mRNA recognition by HuR.

RRM3 may stabilize the RRM1–2 complex [46] and, together with the hinge region, might be important for interacting with other proteins [46], as well as for HuR dimerization [47] and multimerization in cancer cells [48]. In vivo tested ligands, such as dehydromutactin [29], okicenone [29], triptolide [33], leptomycin B [34], and selinexor [35], have been suggested to bind to RRM3 (or the hinge region), thereby hindering HuR translocation to the cytoplasm [44].

Despite various laboratories initiating programs to develop inhibitors of HuR, no FDA-approved drug for HuR has been reported so far. Characterizing efficiency and selectivity of the above-mentioned inhibitors has turned out to be a challenging task [49], mostly because not only does HuR interact with a complex network of proteins, but it is also heavily post-translationally modified [50,51]. Such interplay between modifications and cellular partners determines HuR’s subcellular localization and disease-related dysregulation, and, ultimately, is responsible for some inhibitors to only be effective in specific experiments and/or cell lines, increasing the chance of false-positive results [49]. This is the case for MS-444, the only compound that has been tested in preclinical models targeting HuR so far [40]. This compound is active against cytoplasmic, but not nuclear, HuR [40], suggesting that MS-444 specificity requires additional investigation. Furthermore, MS-444, as well as other inhibitors, like DHTS and CMLD-2, were only tested against specific types of cancer (including colorectal cancer, adenocarcinoma, and glioma), while their effect on other HuR-associated pathological conditions, like inflammation or neurological disorders, is currently unknown [49]. Therefore, the identification of HuR inhibitors and the rationalization of their inhibition mechanisms, based on the HuR structure and independently from specific disease states, is still open to potential breakthroughs. In this regard, a rational structure-based approach for the identification of such ligands, which is so far lacking, would be of great help to reach this goal.

This is one of the objectives of this paper. Notably, we have used computational methods to identify novel small molecules acting as HuR inhibitors, which were then successfully validated through experiments. Given the lack of ‘enzymatic-like’ binding cavities in RNA-binding proteins, such as HuR [52], we exploited the aforementioned transient binding cleft between RRM1–2 to conduct a structure-based virtual screening combined with RNA pulldown assays. Specifically, we pinpointed key molecular hotspots for HuR inhibition, in agreement with previous NMR and fluorescence polarization assays of known inhibitors [23,24,26,53,54]. We also established a proof-of-concept of our structure-based approach by identifying a dose-dependent inhibitor of HuR with a chemical scaffold different from those reported so far. This is 4-(2-(2,4,6-trioxotetrahydropyrimidin-5(2H)-ylidene)hydrazineyl)benzoate (known as STK018404 hereafter), which is already known as a potential antibacterial and antiviral agent (PubChem CID 942573). The approach used here may be now applied to design highly needed ligands targeting HuR against neurological disorders and inflammatory diseases.

## 2. Results

### 2.1. Screening

**Figure 2:**
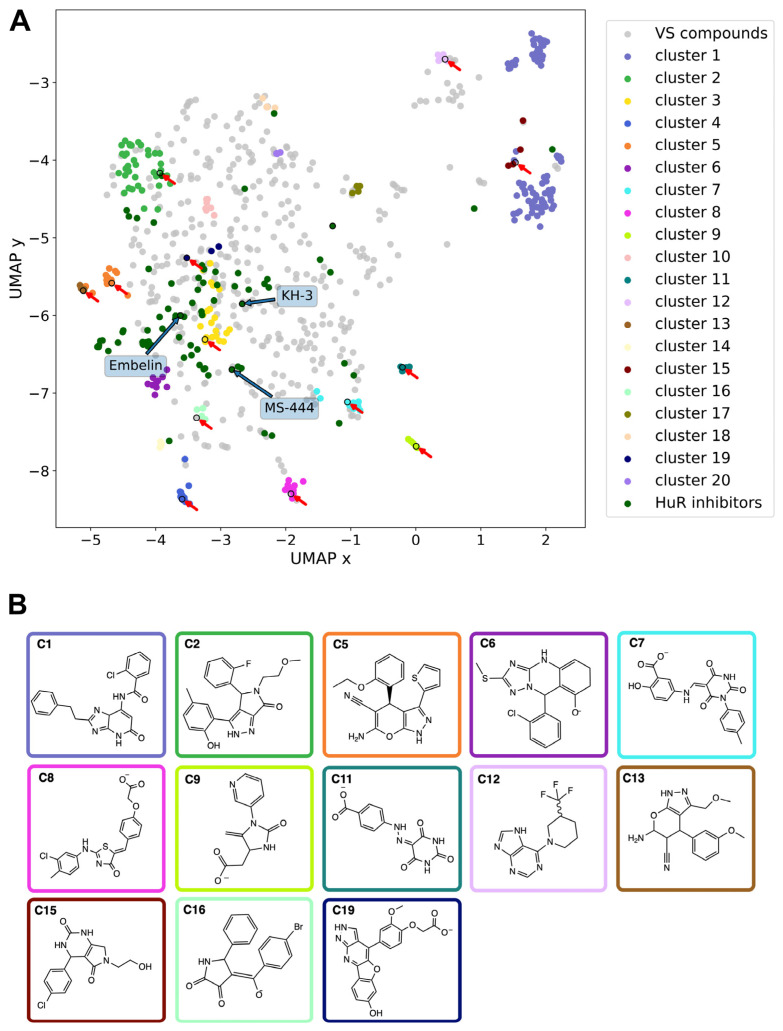
Chemical features of HuR ligands. (**A**) Representation of the chemical space occupied by the 1221 hits identified via virtual screening and 70 HuR inhibitors previously published as of January 2023 (see Table S1). Blue and red arrows indicate previously reported in vivo HuR inhibitors (see Figure S1) and hit molecules identified and tested experimentally in the present study, respectively. (**B**) Chemical structures of the compounds purchased for the thirteen HTVS clusters commercially available; the box colors indicate the corresponding clusters in (**A**). Representative, member, and derivative indicate whether the compound corresponds to the cluster representative, a member of the cluster, or a derivative compound. (C1) derivative, Z259632494; (C2) derivative, Z57908816; (C5) representative, 7643436; (C6) derivative, STK217448; (C7) member, STL170802; (C8) derivative, 5974204; (C9) member, Y044-6405; (C11) representative, STK018404; (C12) derivative, Z373002224; (C13) derivative, STK806137; (C15) derivative, STK593921; (C16) derivative, STL148963; and (C19) representative, STL522523.

We performed a structure-based virtual screening against the binding site previously identified via NMR for other small molecule HuR inhibitors [53], namely the above defined transient binding cleft between RRM1 and RRM2. The high-throughput virtual screening (HTVS) tool in Maestro from Schrödinger software (version 13.5.128) [55,56] was used (see Section 4 for details), together with the following libraries: KIT (11,046 ligands), ENAMINE (200,057), and MOLPORT (8,084,644), for a total of approximately 8.3 million compounds. The screening resulted in 1221 hits (Figure 2A), which were clustered according to the Butina algorithm [57], with Daylight fingerprints and Tanimoto similarity scores [58] and using a 0.3 cutoff (see Section 4 for further details). We then selected the 20 most populated clusters (Figure S2) and identified a representative molecule for each of them (Figure S3). Out of these twenty clusters, seven (clusters 3, 4, 10, 14, 17, 18, and 20) only contained compounds that were not available for purchase and thus could not be experimentally tested. Compounds belonging to the remaining thirteen clusters (Figure 2B) were selected for experimental testing based on their commercial availability. Specifically, the representatives of clusters 5, 11, and 19 were purchased directly. For clusters 7 and 9, a member of the respective cluster was bought. For clusters 1, 2, 6, 8, 12, 13, 15, and 16, a derivative structure of the cluster representative was purchased.

### 2.2. Comparison with the Chemical Space of the Existing Ligands

The chemical space of the HTVS-identified hits was analyzed and compared with previously experimentally validated molecules targeting the RRM1–RRM2 binding cleft (hereafter called HuR inhibitors, see Table S1). To this aim, a non-linear dimensionality-reduction technique, termed uniform manifold approximation and projection (UMAP) [59], was employed. As shown in Figure 2A, the molecules belonging to the 20 most populated clusters spanned a broader chemical space compared to the known inhibitors. Nonetheless, there is an overlap between the inhibitors and HTVS clusters 1, 2, 3, and 6, whereas clusters 7, 10, 14, and 16 are still in the vicinity of the inhibitors. In contrast, cluster groups 4, 8, 9, 11, 12, and 19 occupied distinct parts of the chemical space and consist of novel chemotypes when compared to previously reported HuR inhibitors (Figure 2A).

**Figure 3:**
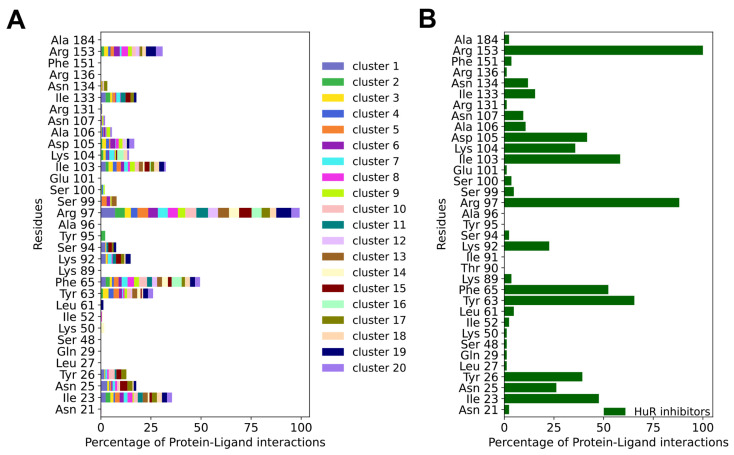
(**A**) Averages of the protein–ligand interaction fingerprints (PLIFs) of the representative compounds of the HTVS clusters identified in this study vs. (**B**) PLIFs of previously reported HuR inhibitors. The normalization was performed between the maximum and the minimum number of interactions for each PLIF. Specifically, in (**A**) PLIFs were normalized using the number of cluster members for each cluster and to the maximum number of interactions shared between all compounds (Arg 97), whereas in (**B**) PLIFs were normalized to the maximum number of interactions shared between all inhibitors (Arg 153). The docking scores of the 13 experimentally tested ligands and the known HuR inhibitors are reported in Table S2.

Mapping the protein–ligand interaction fingerprints (PLIFs) of each cluster group (stacked bar plot in Figure 3A and Figure S4) showed no significant difference among the HTVS hits, despite their unique chemotypes. Comparing the PLIFs of the HTVS compounds (Figure 3A) with the previously reported inhibitors (Figure 3B), similar binding residues were identified. The key common residues are Ile23, Tyr63, Phe65, Arg97, Ile103, and Arg153, as they lie in the top six of the most contacted residues in both panels A and B. Interestingly, Ile23, Arg97, Ile103, Ile133, and Arg153 have been shown to participate in HuR binding to known inhibitors, as well as mRNA, via NMR and fluorescence polarization assays combined with mutagenesis [20,45,54].

For the HuR inhibitors, additional high-frequency contact residues are Asn25, Tyr26, Lys92, Ser94, Lys104, Asp105, Ala106, Asn107, and Asn134. Two thirds of these residues were shown to form interactions with the RNA backbone (PDB 4ED5) [45], namely Asn25, Tyr26, Lys92, Lys104, Asn107, and Asn134. This is not unexpected, considering that HuR inhibitors aim at competing with mRNA binding.

### 2.3. Experimental Test

**Figure 4:**
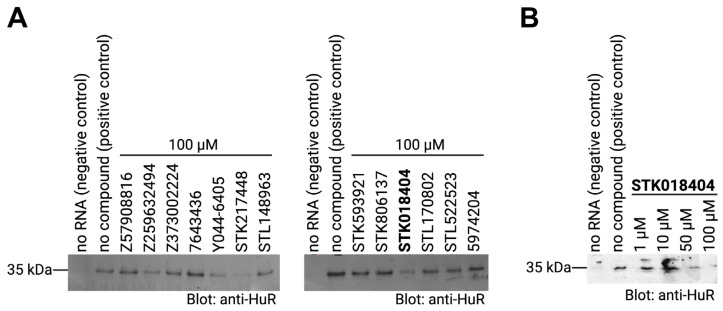
(**A**) RNA–protein pulldown of HuR with its target RNA motif (AUUUUUAUUUU) in the absence (positive control) or presence of the respective compounds (final concentration: 100 µM). RNA-bound proteins were analyzed on western blots detecting HuR. A negative control that does contain RNA and a positive control with no compound were included. (**B**) RNA–protein pulldown of HuR as described in (**A**) with different concentrations of the compound STK018404. The dose-response gels for compounds Y044-6405 and STK217448 are reported in Figure S5.

As mentioned in the introduction, the HuR protein binds its target mRNAs through AU-rich elements. Thus, we used an AUUUUUAUUUU sequence (as shown in Figure 1B) to test whether the predicted compounds affect the binding between the HuR protein and its target mRNA by performing RNA–protein pulldown assays. In particular, biotinylated RNA oligos comprising the HuR-binding motif were incubated with cell extracts that contained the HuR protein in the presence or absence of the compounds. RNA–protein complexes were then purified with streptavidin-coated beads and RNA-bound proteins were analyzed by western blotting using an antibody detecting HuR. In addition, an experiment without RNA was performed as a negative control. As expected, a strong band of the HuR protein alone was detected in the sample with no compound (positive control). Out of the thirteen compounds that we tested, three compounds, Y044-6405, STK217448, and STK018404, decreased the binding of HuR to its target RNA motif (Figure 4A). In a second experiment testing different doses of these three compounds, only STK018404 showed a robust, dose-dependent effect on the binding of HuR to its target RNA motif (Figure 4B).

### 2.4. Docking and Fingerprints

The most promising compound according to the in vitro experiments is the representative of cluster 11, STK018404 (Figure 2B). When compared to previously reported HuR inhibitors, this small molecule is composed of novel chemotypes, namely barbituric acid and benzoic acid fused via a diazo bridge.

**Figure 5:**
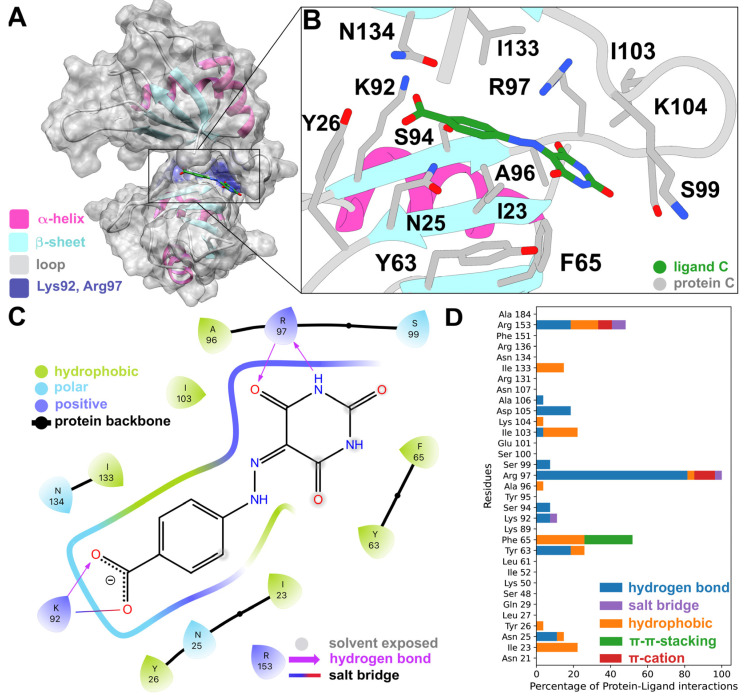
(**A**) Compound STK018404 in the HuR RRM1/2 transient binding cleft. (**B**) Docking pose, in licorice fashion, of compound STK018404 with labeled residues within 4 Å of the ligands. The protein backbone is displayed as a cartoon representation, with the same color code as panel (**A**). (**C**) Corresponding PLIF as a 2D interaction diagram. The color of the residues indicates their physicochemical properties, whereas the colorful thick line represents the binding cavity, with gaps indicating solvent exposure of the ligand. (**D**) PLIFs of the other tested molecules as a bar plot. For the sake of completeness, the PLIF of each of the other twelve tested compounds is shown in Figure S6.

Our docking calculations predicted that it binds to a part of the binding cleft near the linker (Figure 5A,B). It interacts with Arg97 via two hydrogen bonds and with Lys92 via a salt bridge and a hydrogen bond (Figure 5C). When compared to the PLIFs of the other 12 compounds we tested experimentally, Arg97 was also identified as the most contacted residue, especially via hydrogen bonds (Figure 5D). Five residues (Ile23, Phe65, Ile103, Lys104, and Ile133) only showed hydrophobic interactions, with Phe65 also displaying pi–pi-stacking interactions. Other hydrogen-bonded residues were Arg153, Lys92, and Tyr63. π-cation interactions were only seen for Arg97 and a small number of salt bridges for Arg97 and Arg153. Out of the nine residues predicted to form interactions with STK018404, four have been shown via NMR to interact with other HuR inhibitors (Arg97, Ile103, Ile133, and Arg153) [24,53,54], whereas three others (Phe65, Lys92, and Lys104) are known to participate in RNA binding by X-ray crystallography [45].

While eleven of the compounds tested experimentally here interact mainly with the linker (residues 96–103 in Figure S5), the remaining two compounds, STK018404 and STL170802 (representing clusters 11 and 7), extended further into the RNA binding cleft (Figure S7). The rigid scaffold and carboxylate moiety present in both these molecules (CR11 and CR7 in Figure 2B, respectively) allow for the simultaneous formation of hydrogen bonds with the backbone of Arg97 and contacts with Lys92 (via a hydrogen bond and a salt bridge). However, STL170802 has an additional hydrophobic toluene ring that, according to our docking model, was mostly exposed to the solvent. This might be responsible for a less stable binding pose and, as a result, the reduced inhibitory effect in HuR binding of RNA.

### 2.5. Off-Targets Prediction

Possible off-targets for our HTVS ligands (along with representatives of the previously known HuR inhibitors, shown in Table S1 and Figure S1) could, in principle, be evaluated by docking our top hits against these off-targets and by comparing the docking scores with those found for HuR. However, unfortunately, using this approach is highly non-trivial, since neither off-targets of HuR inhibitors are known experimentally (to the best of our knowledge) nor they cannot be identified computationally in a straightforward manner. Indeed, on the one hand, historically, RNA recognition motifs (RRMs), like the ones present in HuR, were considered as ‘undruggable’ domains for a long time [22,30]. On the other hand, the most straightforward prediction, which would exploit the sequence similarity between the target binding pocket(s) and other biomolecules’ binding sites [60,61], is not possible here. While the overall structure of the RRMs present in HuR and other RNA-binding proteins (RBPs) is highly conserved, the position and conformation of the RNA at the surface of the RRM complexes is highly variable, as well as the exact binding site on the RBPs [62]. This diversity is due to three main reasons. First, in multi-RRM proteins, the inter-domain linkers present different lengths, conformations, and orientations relative to the RRM domains. Yet, this linker participates in the RNA binding of most RNA–RRM complexes. Second, the relative orientation of the RRMs in the complex is variable. Finally, the sequence identity among the RRMs is quite low (typically 20–30%). Thus, protein sequence similarity approaches would not be effective here.

In an effort at addressing this important issue, we predicted off-targets by establishing whether our identified hit molecules are chemically similar to known ligands binding to highly common human protein targets. To achieve this aim, we used the SwissTargetPrediction web server (http://www.swisstargetprediction.ch/, accessed on 8 August 2023) [63]. This webtool compares the molecules of interest against a set of about 370,000 ligands, known to be active against about 3000 proteins.

No significant match or low similarity with known active molecules results in ‘no off-target found’ or off-targets with a 0–15% predicted probability, respectively. This might point to a greater potential of the analyzed molecule to be specific against the primary target (here HuR).

The compound in preclinical tests, MS-444 (along with a few other in vivo ligands, e.g., okicenone and triptolide), features non-significant similarity with the active ligands present in the SwissTargetPrediction database; thus, no off-target could be found. Among the HuR ligands tested either in vivo or in cellulo, quercetin features a probability above 80% to bind up to 69 different off-targets. The same high probability was found for CMLD-2, DHTS, mitoxantrone, hyperoside, and chlorogenic acid, with one to fifteen off-targets predicted. The other remaining representatives of the known HuR inhibitors exhibited a 0% or below 15% probability to bind off-targets (with 20 to 100 protein candidates reported).

In contrast, almost all of our tested molecules (i.e., 12 out of 13) displayed either no significant matches with known active molecules or probabilities below 15% to bind off-targets. In particular, our most promising compound, STK018404, features only seven possible off-targets (among which include the oxidoreductase, transferase, and kinase protein classes) with low probabilities between 10% and 15%. The other-top ranked 93 off-targets displayed a 0% probability of binding.

Altogether, our results further support the power of our approach in identifying novel scaffolds and the potential of our hit molecule to be more specific than several other known HuR inhibitors. However, these considerations must be taken with caution since the SwissTargetPrediction database is biased toward active molecules against the most common ‘druggable’ targets in the CHEMBL23 database. Ligands targeting RRMs, which were considered “undruggable” for a long time [22], are still dramatically under-represented here (and in other pharmacological databases) [64,65].

## 3. Discussion

RNA-binding proteins (RBPs) are a protein class that, historically, have not been extensively targeted using small molecule approaches [22]. In the past few years, however, several successful examples of small molecules targeting RBPs have highlighted their potential for therapeutic applications in various diseases [22].

HuR is a master post-transcriptional regulator that is mainly involved in messenger RNA (mRNA) translation and turnover [1]. Therefore, HuR is a potential therapeutic target of wide impact for several diseases, including cancer, inflammation, cardiovascular, muscle, kidney, and liver diseases [49]. Several small molecules have been identified, including MS-444 [40], which is currently being tested in preclinical models.

Here, the application of virtual screening approaches provided access to an enlarged chemical space of molecules potentially targeting HuR and impairing RNA binding. Among the selected molecules, experimental validation identified the ligand STK018404 as a promising HuR inhibitor, with a dose-dependent impairment of RNA binding against the full-length protein. Our docking calculations predicted that it binds to the transient binding cleft (Figure 5B), which also accommodates cognate mRNA nucleobases and other small ligands [24]. STK018404 forms two hydrogen bonds with Arg97 and a hydrogen bond and a salt bridge with Lys92 (Figure 5C). These two residues are also known to interact with a variety of other ligands, including CMLD-2, hyperoside, rutin, isovitexin, novobiocin, chlorogenic acid, 7-hydroxymatairesinol, colchicoside [24,54], and azaphilone-9 [53], as well as uracil nucleotides of the RNA (U8 and U9 in PDB 4ED5) [45]. However, its scaffold (Figure 5B) differs largely from those of the ligands that have been identified so far (Figure S1 and Table S1), providing new endeavors for future drug design campaigns.

In particular, this holds out the possibility to identify novel binding modes or chemical structures with a higher selectivity compared to the already available HuR inhibitors, as well as being an important factor for potential patentability [66].

Furthermore, our approach was able to rationalize and highlight the common binding features across HuR inhibitors, and their shared key molecular anchoring points on HuR, independently of their chemical scaffolds.

In vivo assays could assess whether STK018404 encounters issues of specificity and potency, as it is the case for several other small molecules targeting HuR [29,32,33,35,36,37,40].

## 4. Materials and Methods

### 4.1. Experimental Methods: RNA Pulldown

A biotinylated RNA oligo comprising the HuR-binding motif (biotin-TEG-5′AUUUUUAUUUU-3′ from IDT Integrated DNA Technologies) was dissolved in a RNA structure buffer (10 mM Tris pH 7, 10 mM MgCl2, 100 mM KCl) at a final concentration of 100 pmol/µL = 100 µM, incubated for 10 min at 72 °C, and cooled down slowly to RT. To coat streptavidin-agarose beads (Sigma, St. Louis, MO, USA) with the RNA, 300 pmol of RNA oligo were added to 60 µL beads in 300 μL of Buffer D (20 mM Tris pH 7.9, 20% glycerol, 0.1 M KCl, 0.2 mM EDTA, and 0.5 mM DTT) and incubated for 30 min at RT.

Following this step, 4 × 106 HEK293 T cells were seeded in a 150 cm^2^ flask (cell binding surface), cultured for 48 h, and harvested using a cell scraper. The cells were lyzed in 1 mL of Buffer D containing a RNAse inhibitor (RiboLock) using sonication. Cell debris was removed through centrifugation for 10 min at 12,000× *g* at 4 °C. After pre-clearing, the protein lysate was incubated with the RNA-coated beads at 4 °C overnight in the presence or absence of the compounds. After extensive washing, RNA-bound proteins were eluted by boiling the beads in 30 μL 3× Lämmli buffer (150 mM Tris-Cl pH 6.8, 6% SDS, 30% glycerol, 15% ß-mercaptoethanol, and 0.3% bromophenol blue) for 5 min at 95 °C. RNA-bound proteins were then analyzed on western blots using anti-HuR antibodies (Santa Cruz, Dallas, TX, USA, SC5261) to detect HuR.

Uncropped western blot gels corresponding to the RNA pulldown assays shown in Figure 4 have been reported in Figure S8.

### 4.2. Computational Methods

#### 4.2.1. Structure-Based Virtual Screening

The HuR protein structure (PDB code: 4ED5 [45]) was preprocessed using the Protein Preparation Wizard from the Maestro Schrödinger Suite version 2022-1 with default parameters [67]. The protonation states of each side chain were generated using Epik for pH = 7 ± 2 [68]. Protein minimization was performed using the OPLS3 force field [69]. All water molecules were removed. Virtual screening studies were then performed with the HTVS [55,56] feature in Maestro using a repurposing library, including all the commercialized and under development ligands retrieved from the Molport (8,084,644 molecules), Enamine (200,057), and KIT (11,046) libraries. The ligands were docked using a receptor grid, encompassing the RNA binding cleft of the HuR RRM1/2–RNA complex, as used in previous studies [23,24,36,54]. Twenty poses were generated for each ligand. The employed docking scoring function was Glidescore (GScore), an empirical function that is able to reproduce trends in the ligand binding affinity to the same protein target, which has been defined by the following equation:

GScore = a × vdW + b × Coul + Lipo + Hbond + Metal + Rewards + RotB + Site(1)

where vdW—van der Waals interaction energy; Coul—Coulomb interaction energy; Lipo—lipophilic contact plus phobic-attractive term; HBond—hydrogen-bonding term; Metal—metal-binding term; Rewards—various reward or penalty terms; RotB—penalty for freezing rotatable bonds; and Site—polar interactions in the active site; the coefficients of the vdW and Coul terms are: a = 0.050 and b = 0.150 for Glide 5.0. The contribution from the Coulomb term was capped at −4 kcal/mol. The HTVS-based screening resulted in 1221 hits (Figure 2A). Afterwards, Lipinski and reactive group filtering was performed in Maestro Schrödinger. This allowed to remove compounds with undesirable properties and to focus on drug-like hits, leaving 639 compounds. These were subsequently clustered according to the Butina clustering algorithm [57] in RDKit [70], with Daylight fingerprints and Tanimoto similarity scores [58], using a 0.3 cutoff. We then selected the 20 most populated clusters (Figure S2) and identified a representative molecule for each of them (Figure S3). For the 70 known HuR inhibitors (Table S1) the same docking protocol was utilized and the corresponding docking scores are reported in Table S2. A decoy test was performed to validate the performance of our docking protocol (Figure S9 and Table S5). The decoys were generated with the DUDE-Z webserver [71], using our active hit molecule as the input. The subsequently generated 200 decoy molecules were docked to HuR using the same protocol described above, resulting in twenty-three successfully docked poses. The results show that decoys are indeed recognized as the worst binders with respect to our hit molecule. However, these results must be taken with caution since reliable decoy libraries are usually experimentally validated to be inactive molecules, and this is not the case here.

#### 4.2.2. PLIF Analyses

The docking poses of the molecules belonging to the 20 most populated clusters were, furthermore, used to determine their protein–ligand interaction fingerprints (PLIFs), using the protein–ligand interaction profiler (PLIP) tool [72] to gain insight regarding the key binding residues (Figure S4). The same method was applied for the generation of the protein–ligand interaction fingerprints of the HuR inhibitors found in the literature, as well as for the decoys. The PLIP uses knowledge-based thresholds and geometric criteria to search for the most relevant contacts in the neighborhood of every residue and reports their interaction types [72].

#### 4.2.3. Off-Target Analyses

Clustering of the known HuR inhibitors (Table S1 and references [9,21,23,26,27,28,29,32,33,34,35,36,37,38,39,48,53,54,73,74,75,76]), using the Butina algorithm with Daylight fingerprints and a Tanimoto similarity cutoff of 0.3, resulted in the identification of 11 clusters (with a representative ligand for each), as well as 21 individual compounds belonging to no cluster, for a total of 32 representatives. The off-target analysis was carried out for these 32 HuR inhibitors and our thirteen experimentally tested compounds using the SwissTargetPrediction webserver [63]. As an input to the SwissTargetPrediction webserver, the SMILES string from the best docking pose (see above) was taken. The webserver generated a list of 100 potential protein targets for each ligand with their corresponding probabilities (see Table S3) and provided information about the main protein classes to which these off-targets belong (Table S4).

## Data Availability

The data presented in this study are available upon request from the corresponding author.

## Supplementary Materials

The following supporting information can be downloaded at: https://www.mdpi.com/article/10.3390/ijms241713127/s1.
