# Supplementary material for: Low Molecular Weight Inhibitors Targeting the RNA-Binding Protein HuR
Source: Int J Mol Sci. 2023 Aug 23;24(17):13127. doi: 10.3390/ijms241713127 (PMC10488267; doi:10.3390/ijms241713127)
Supplement: Supplementary file 1 [file ijms-24-13127-s001.zip › ijms-2562015-supplementary.pdf]

International Journal of
Molecular Sciences

MDPI

Supplementary figures & tables

Low molecular weight inhibitors targeting the RNA-binding protein HuR

Benjamin Philipp Joseph [1,2,†], Verena Weber [1,2,†], Lisa Knüpfer [3], Alejandro Giorgetti [1,4], Mercedes Alfonso-Prieto [1], Sybille Krauß [3,*], Paolo Carloni [1,2,*] and Giulia Rossetti [1,5,6]

1 Institute for Neuroscience and Medicine and Institute for Advanced Simulations (INM-9/IAS-5), Computational Biomedicine, Forschungszentrum Jülich, 52425 Jülich, Germany; b.joseph@fz-juelich.de (B.P.J.); v.weber@fz-juelich.de (V.W.); alejandro.giorgetti@univr.it (A.G.); m.alfonso-prieto@fz-juelich.de (M.A.-P.); g.rossetti@fz-juelich.de (G.R.)
2 Faculty of Mathematics, Computer Science and Natural Sciences, RWTH Aachen University, 52062 Aachen, Germany
3 Institute of Biology, University of Siegen, 57076 Siegen, Germany; lisa.knuepfer@uni-siegen.de
4 Department of Biotechnology, University of Verona, 37134 Verona, Italy
5 Jülich Supercomputing Centre (JSC), Forschungszentrum Jülich, 52425 Jülich, Germany
6 Department of Neurology, RWTH Aachen University, 44517 Aachen, Germany
* Correspondence: sybille.krauss@uni-siegen.de (S.K.); p.carloni@fz-juelich.de (P.C.)
† These authors share first authorship as co-first authors.

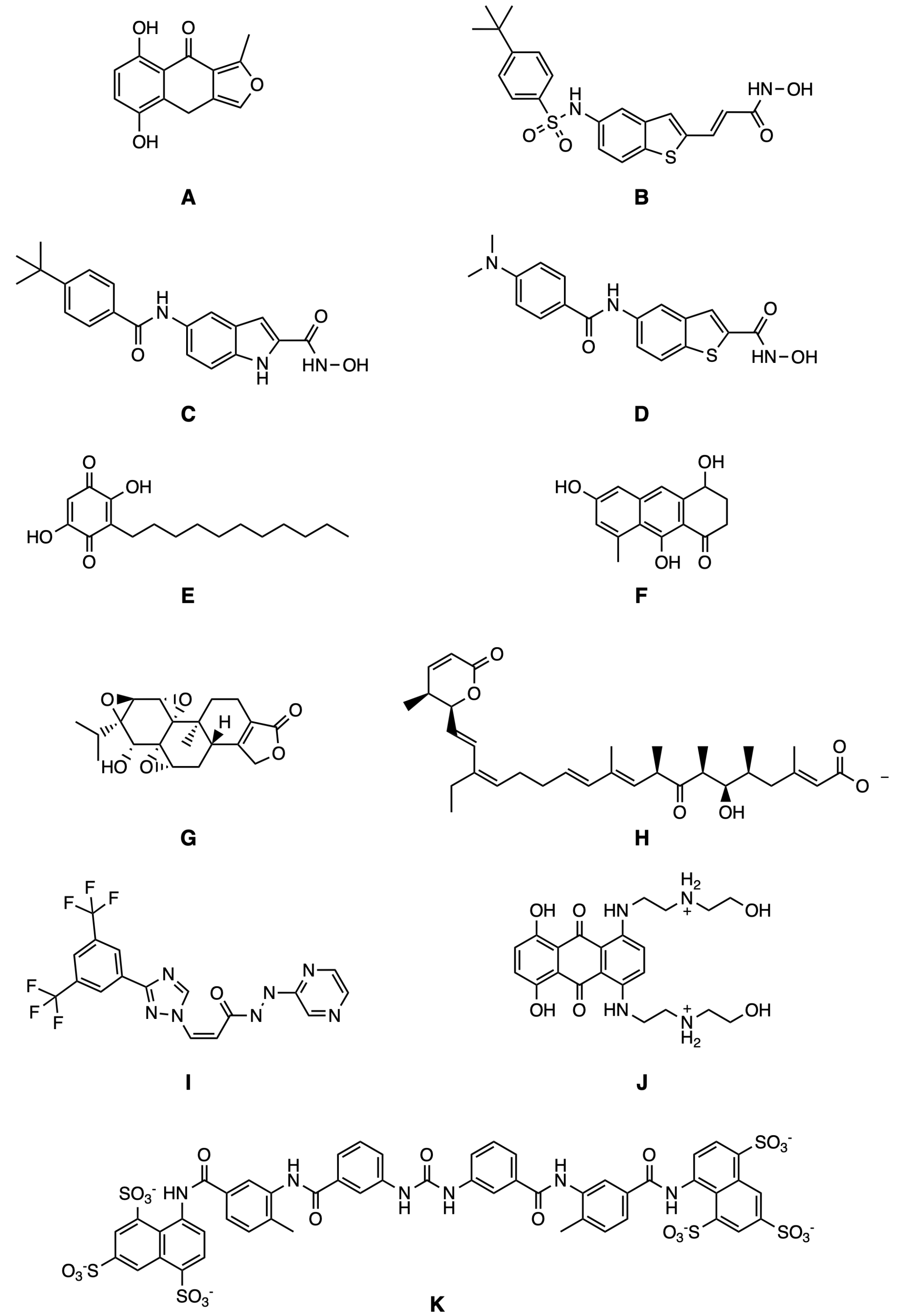

Figure S1. Molecular structures of the HuR inhibitors tested *in vivo*. **(A)** MS-444 (also under preclinical trials). **(B)** KH-3, **(C)** KH-3 derivative 1, **(D)** KH-3 derivative 2, **(E)** Embelin, **(F)** Okicenone, **(G)** Triptolide, **(H)** Leptomycin B, **(I)** Selinexor, **(J)** Mitoxantrone and **(K)** Suramin.

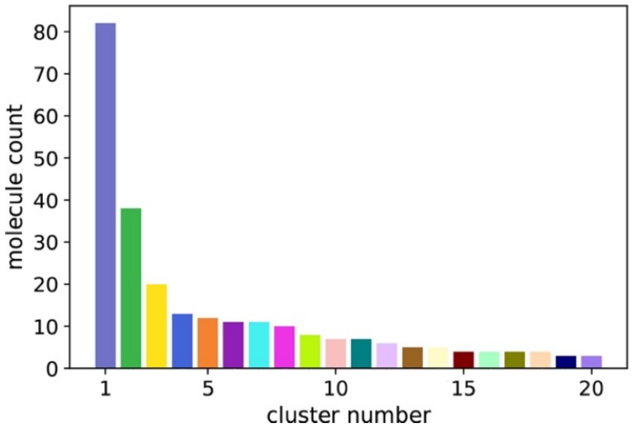

Figure S2. Bar plot representing the number of molecules of the first 20 most populated clusters from the HTVS experiment.

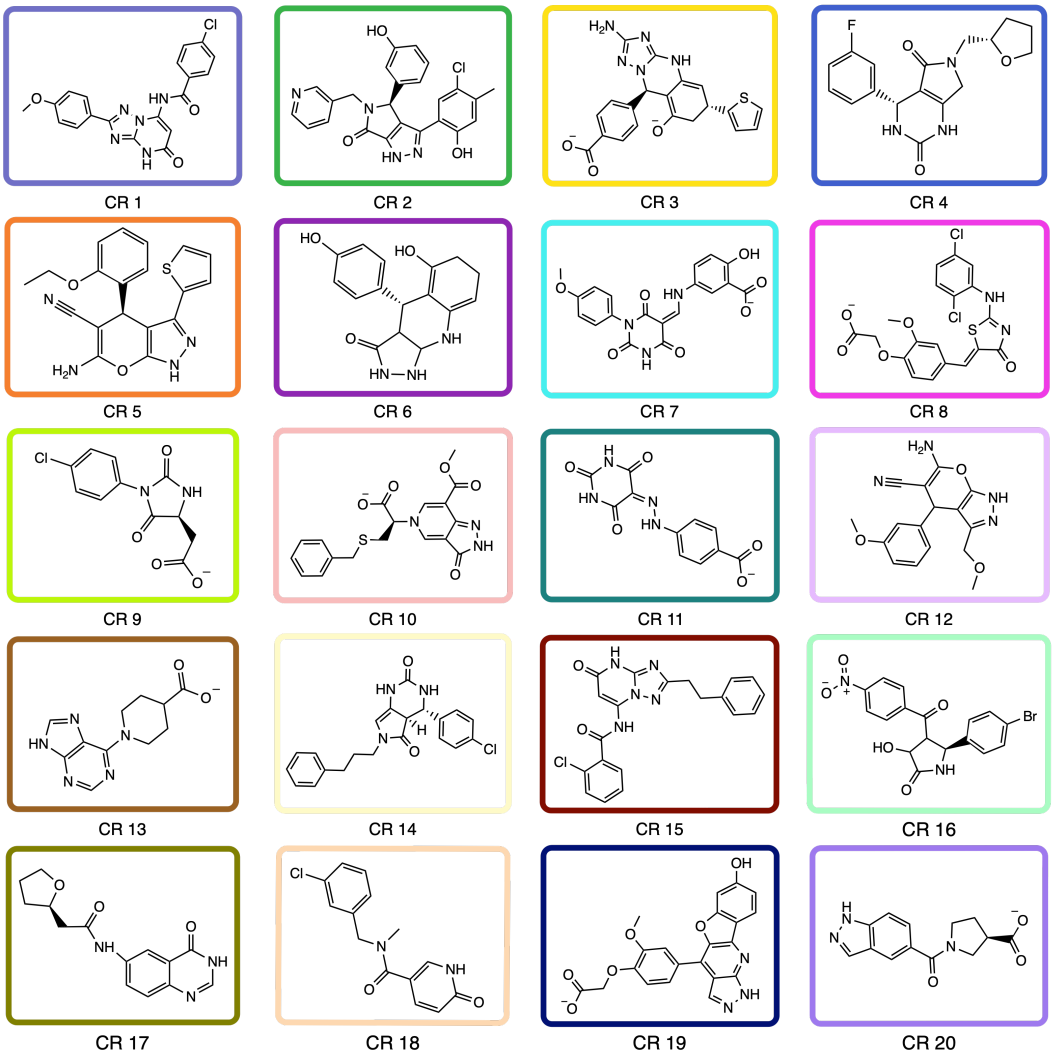

Figure S3. Cluster representatives for the 20 most populated clusters.

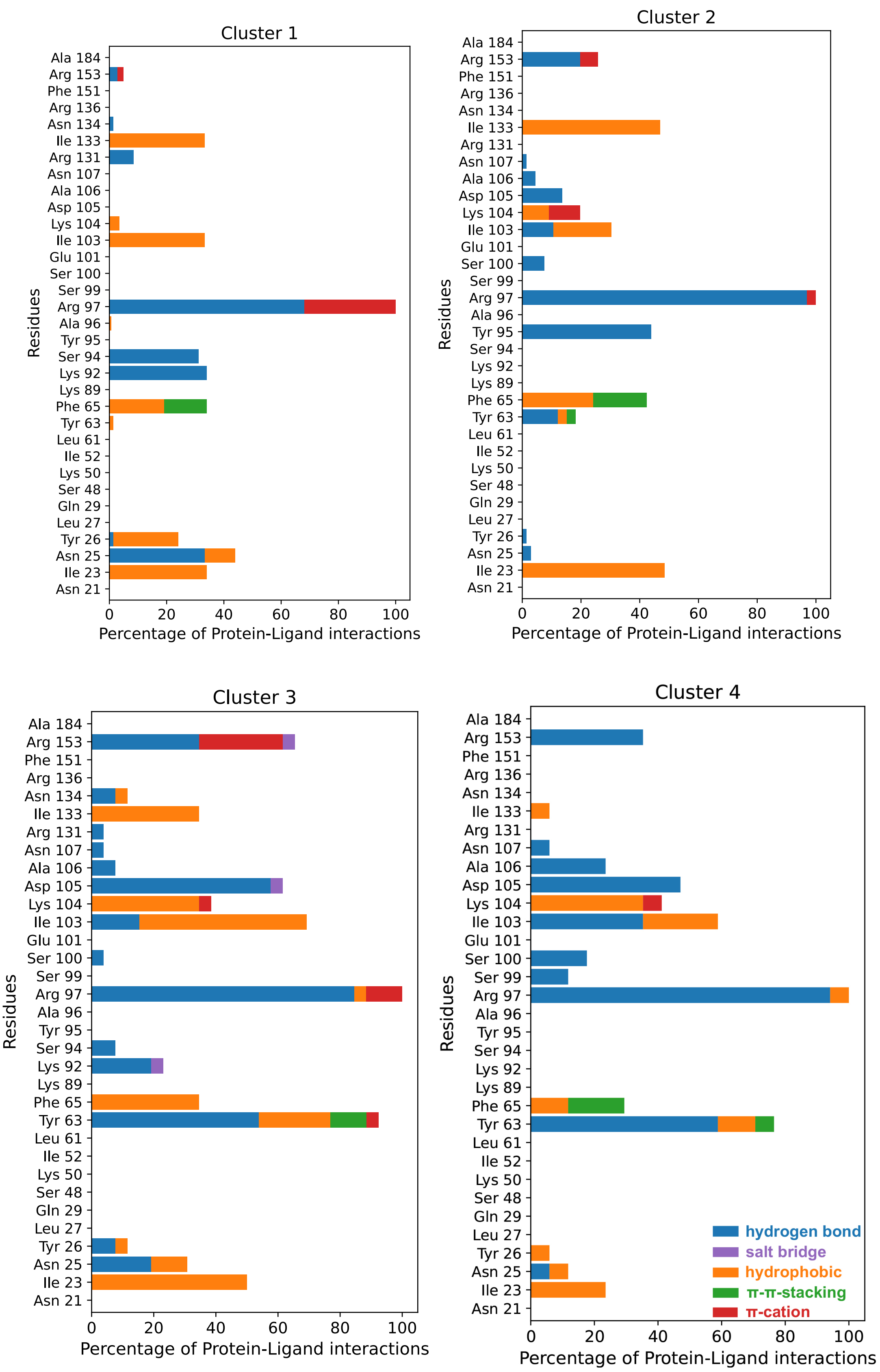

Cluster 1
Cluster 2
Cluster 3
Cluster 4
Residues
Percentage of Protein-Ligand interactions
Ala 184
Arg 153
Phe 151
Arg 136
Asn 134
Ile 133
Arg 131
Asn 107
Ala 106
Asp 105
Lys 104
Ile 103
Glu 101
Ser 100
Ser 99
Arg 97
Ala 96
Tyr 95
Ser 94
Lys 92
Lys 89
Phe 65
Tyr 63
Leu 61
Ile 52
Lys 50
Ser 48
Gln 29
Leu 27
Tyr 26
Asn 25
Ile 23
Asn 21
0
20
40
60
80
100
hydrogen bond
salt bridge
hydrophobic
π-π-stacking
π-cation

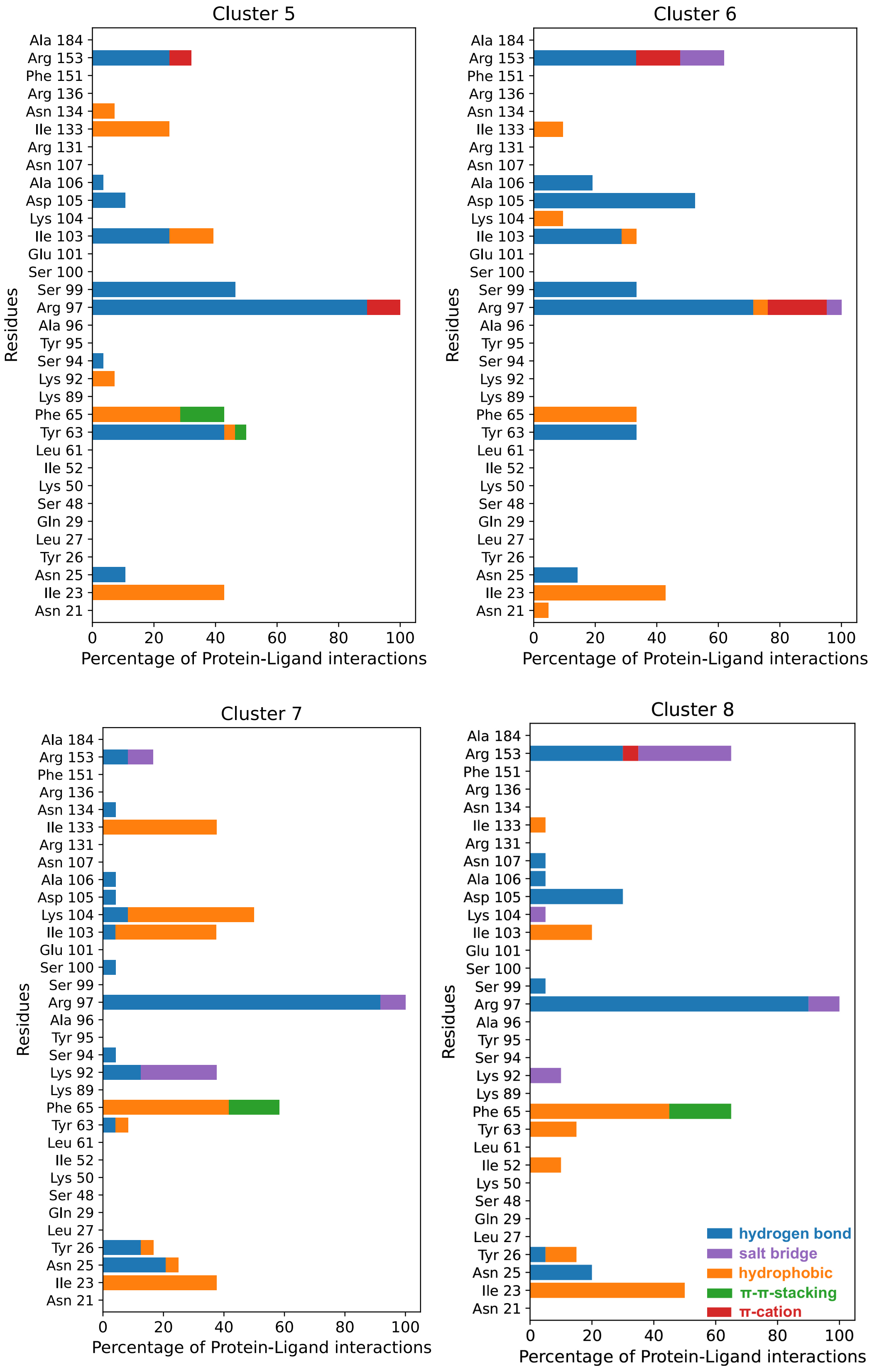

Cluster 5
Cluster 6
Cluster 7
Cluster 8
Residues
Ala 184
Arg 153
Phe 151
Arg 136
Asn 134
Ile 133
Arg 131
Asn 107
Ala 106
Asp 105
Lys 104
Ile 103
Glu 101
Ser 100
Ser 99
Arg 97
Ala 96
Tyr 95
Ser 94
Lys 92
Lys 89
Phe 65
Tyr 63
Leu 61
Ile 52
Lys 50
Ser 48
Gln 29
Leu 27
Tyr 26
Asn 25
Ile 23
Asn 21
0
20
40
60
80
100
Percentage of Protein-Ligand interactions
hydrogen bond
salt bridge
hydrophobic
π-π-stacking
π-cation

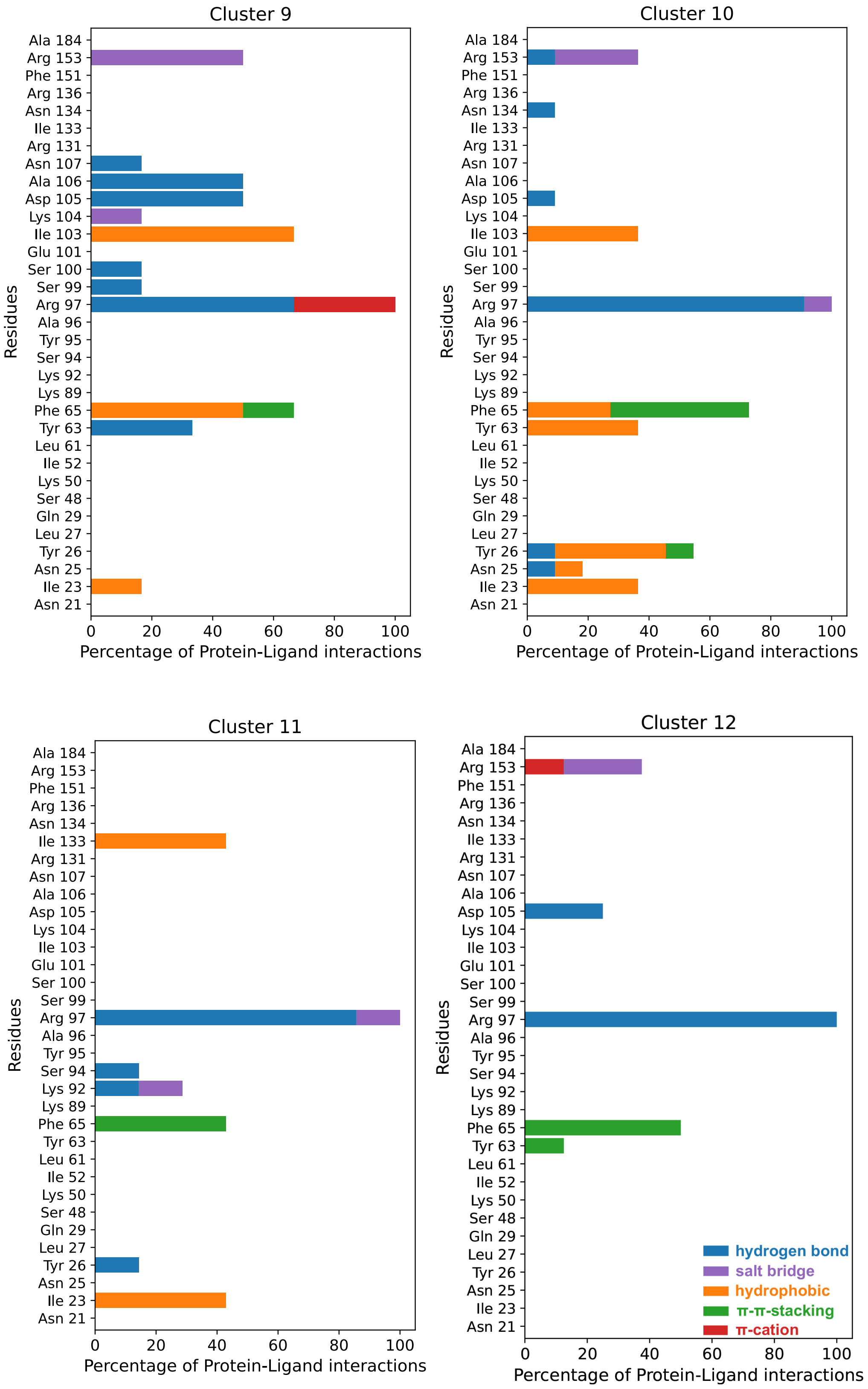

Cluster 9
Cluster 10
Cluster 11
Cluster 12
Ala 184
Arg 153
Phe 151
Arg 136
Asn 134
Ile 133
Arg 131
Asn 107
Ala 106
Asp 105
Lys 104
Ile 103
Glu 101
Ser 100
Ser 99
Arg 97
Ala 96
Tyr 95
Ser 94
Lys 92
Lys 89
Phe 65
Tyr 63
Leu 61
Ile 52
Lys 50
Ser 48
Gln 29
Leu 27
Tyr 26
Asn 25
Ile 23
Asn 21
Residues
0
20
40
60
80
100
Percentage of Protein-Ligand interactions
hydrogen bond
salt bridge
hydrophobic
π-π-stacking
π-cation

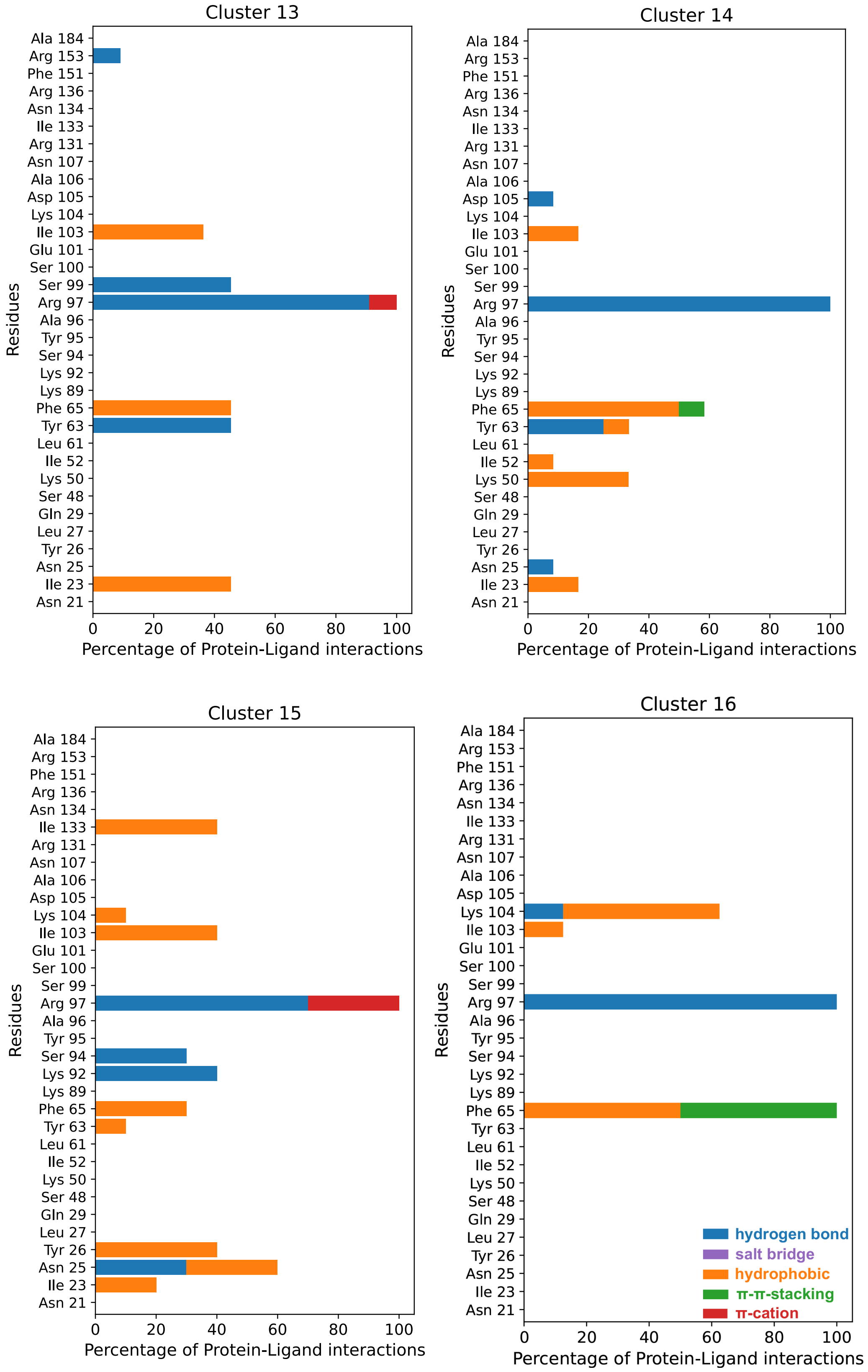
Cluster 13
Cluster 14
Cluster 15
Cluster 16
Residues
Ala 184
Arg 153
Phe 151
Arg 136
Asn 134
Ile 133
Arg 131
Asn 107
Ala 106
Asp 105
Lys 104
Ile 103
Glu 101
Ser 100
Ser 99
Arg 97
Ala 96
Tyr 95
Ser 94
Lys 92
Lys 89
Phe 65
Tyr 63
Leu 61
Ile 52
Lys 50
Ser 48
Gln 29
Leu 27
Tyr 26
Asn 25
Ile 23
Asn 21
0
20
40
60
80
100
Percentage of Protein-Ligand interactions
hydrogen bond
salt bridge
hydrophobic
π-π-stacking
π-cation

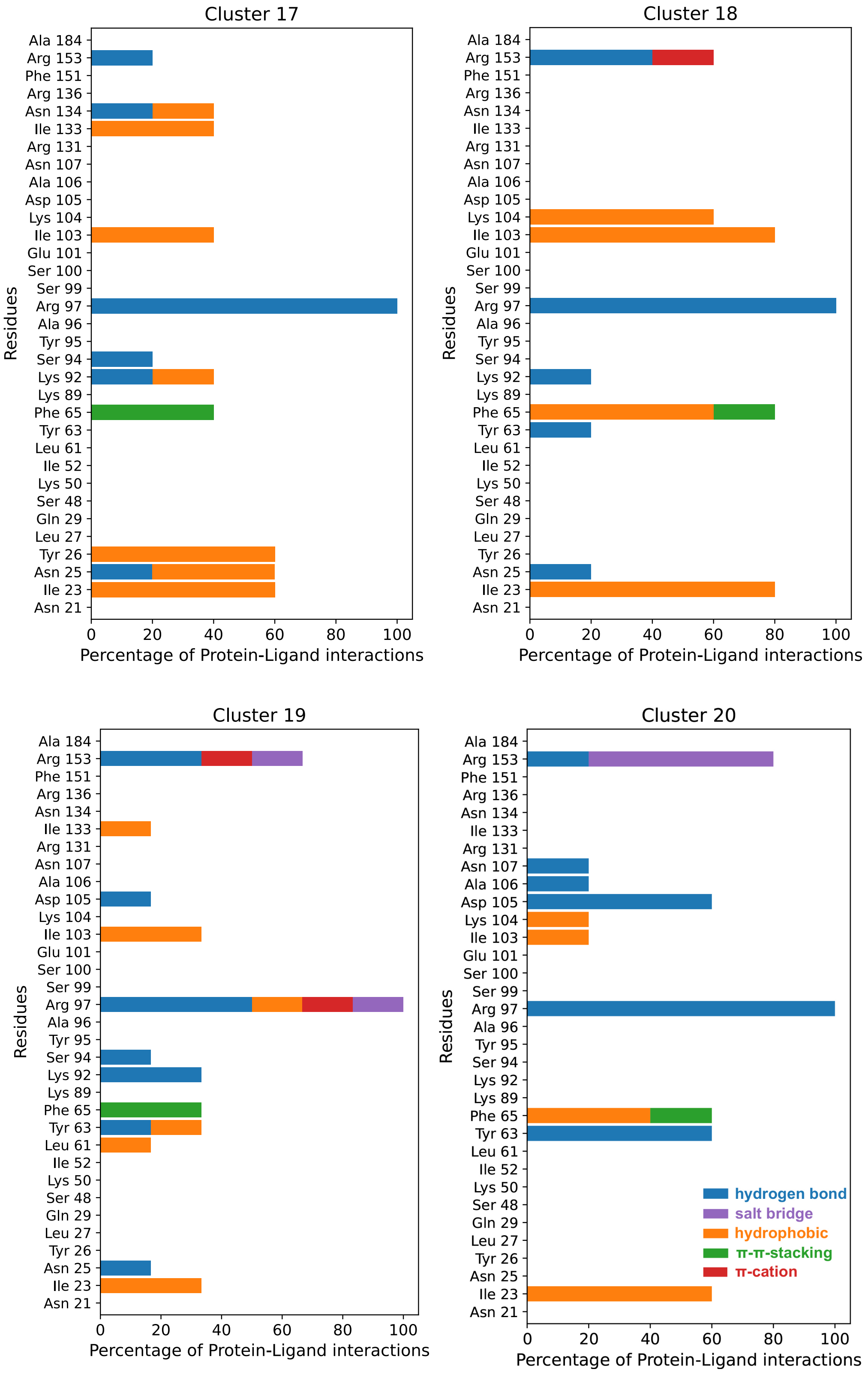

Figure S4. Protein-ligand interaction fingerprints for the 20 most populated clusters.

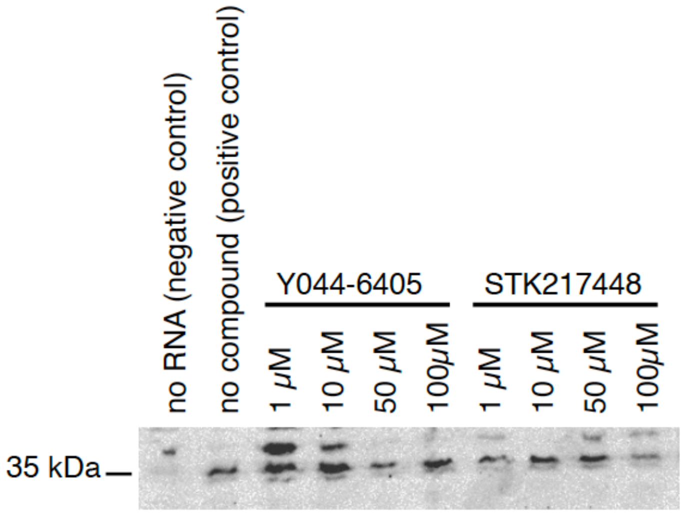

Figure S5. RNA–protein pull-down of HuR as described in Figure 4(A) of the main text with different concentrations of the compounds Y044-6405 and STK217448.

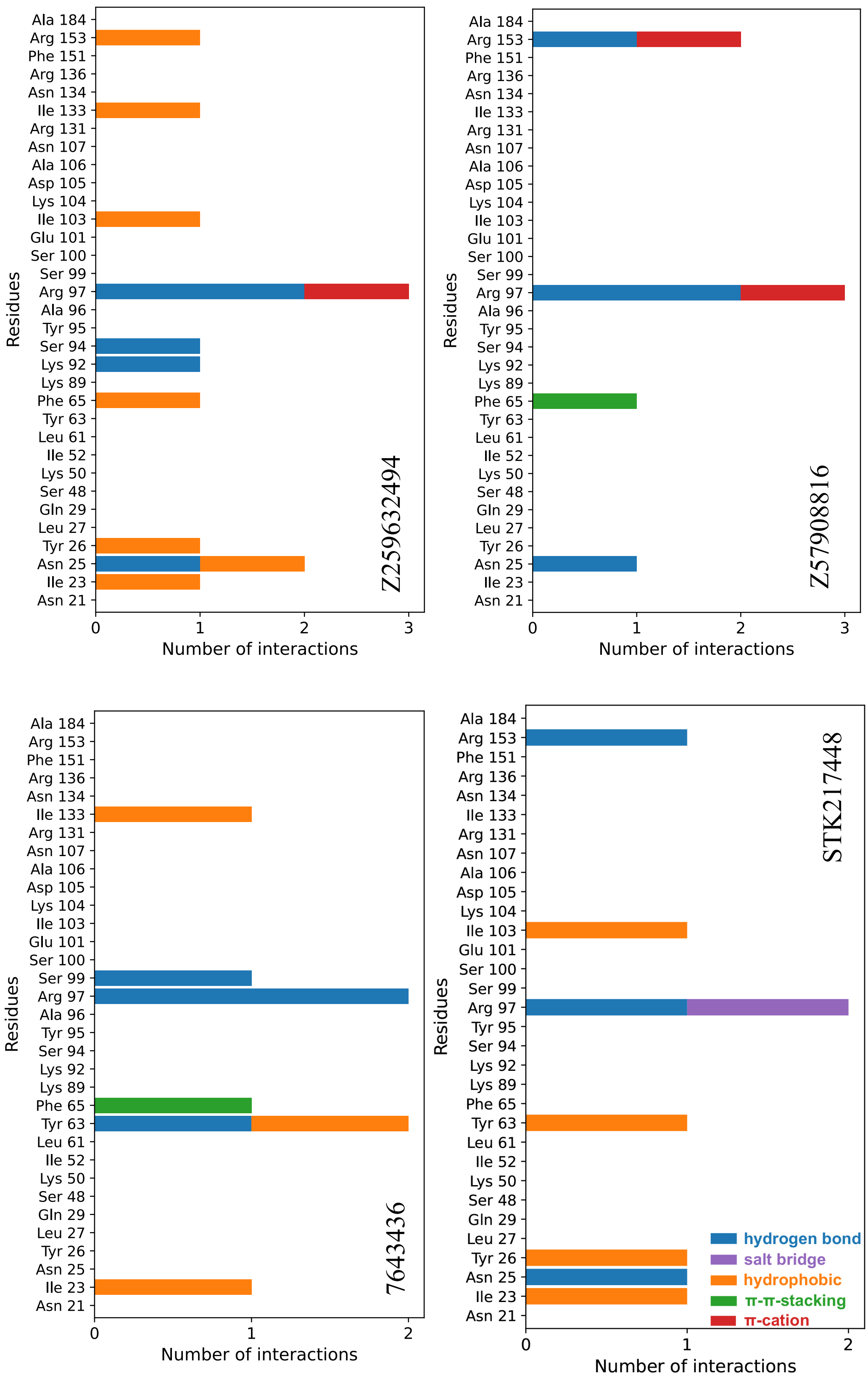
Z259632494
Z57908816
7643436
STK217448
Residues
Number of interactions
Ala 184
Arg 153
Phe 151
Arg 136
Asn 134
Ile 133
Arg 131
Asn 107
Ala 106
Asp 105
Lys 104
Ile 103
Glu 101
Ser 100
Ser 99
Arg 97
Ala 96
Tyr 95
Ser 94
Lys 92
Lys 89
Phe 65
Tyr 63
Leu 61
Ile 52
Lys 50
Ser 48
Gln 29
Leu 27
Tyr 26
Asn 25
Ile 23
Asn 21
hydrogen bond
salt bridge
hydrophobic
π-π-stacking
π-cation

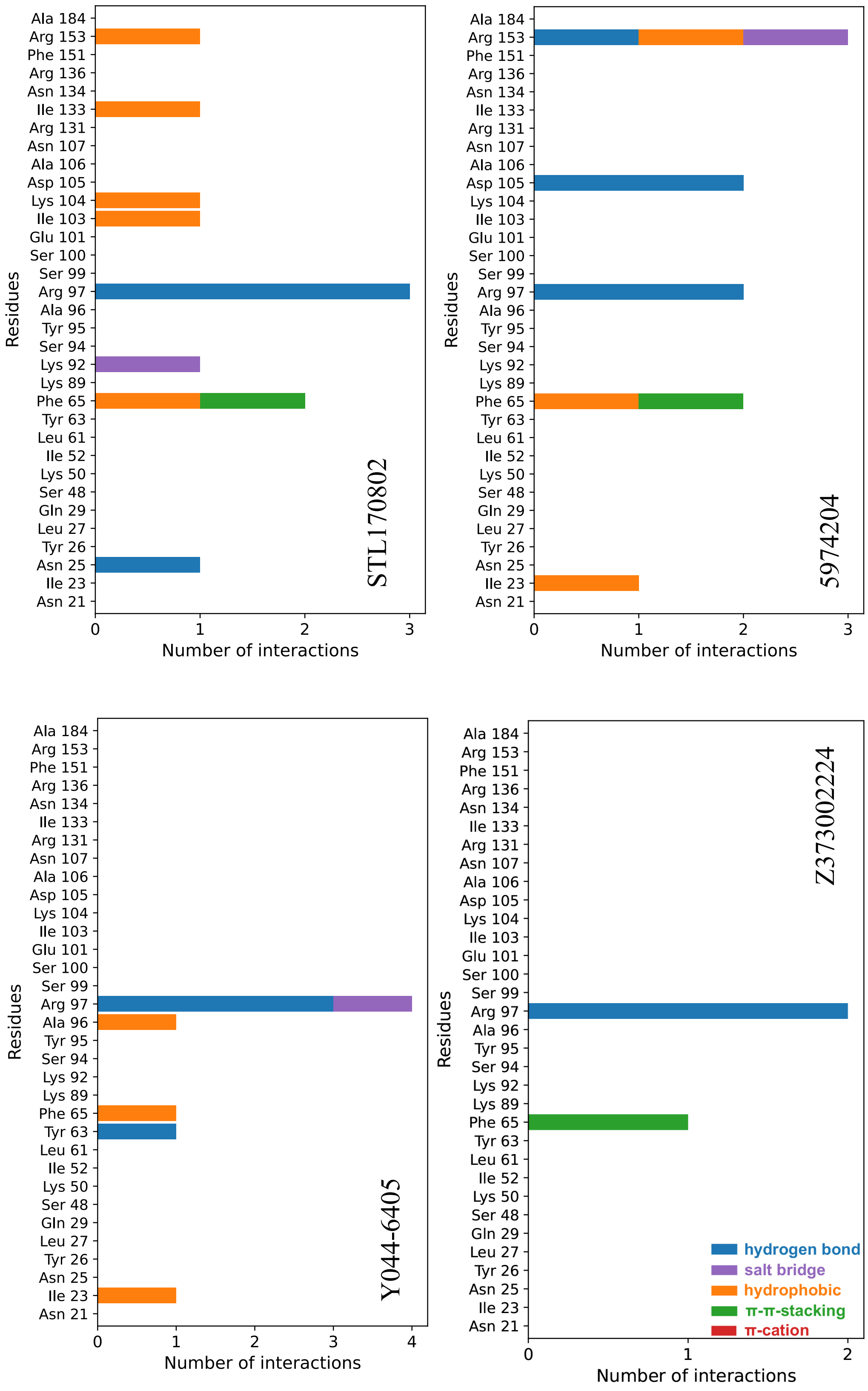
STL170802
5974204
Y044-6405
Z373002224
Residues
Number of interactions
Ala 184
Arg 153
Phe 151
Arg 136
Asn 134
Ile 133
Arg 131
Asn 107
Ala 106
Asp 105
Lys 104
Ile 103
Glu 101
Ser 100
Ser 99
Arg 97
Ala 96
Tyr 95
Ser 94
Lys 92
Lys 89
Phe 65
Tyr 63
Leu 61
Ile 52
Lys 50
Ser 48
Gln 29
Leu 27
Tyr 26
Asn 25
Ile 23
Asn 21
0
1
2
3
4
hydrogen bond
salt bridge
hydrophobic
π-π-stacking
π-cation

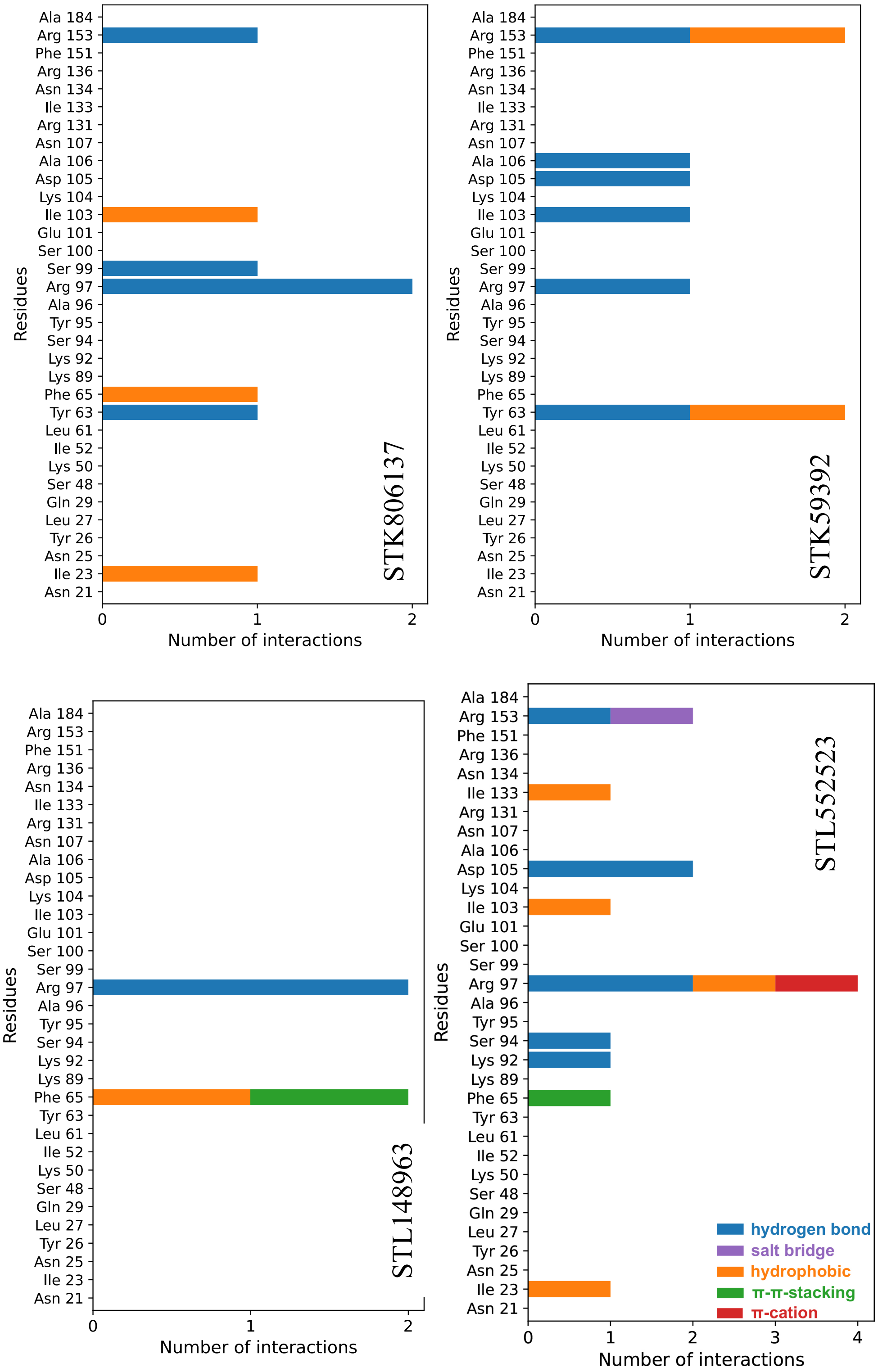

Figure S6. PLIFs of the other 12 bought compounds (C1) derivative, Z259632494; (C2) derivative, Z57908816; (C5) representative, 7643436; (C6) derivative, STK217448; (C7) member, STL170802; (C8) derivative, 5974204; (C9) member, Y044-6405; (C11) representative, STK018404; (C12) derivative, Z373002224; (C13) derivative, STK806137; (C15) derivative, STK593921; (C16) derivative, STL148963; (C19) representative, STL522523.

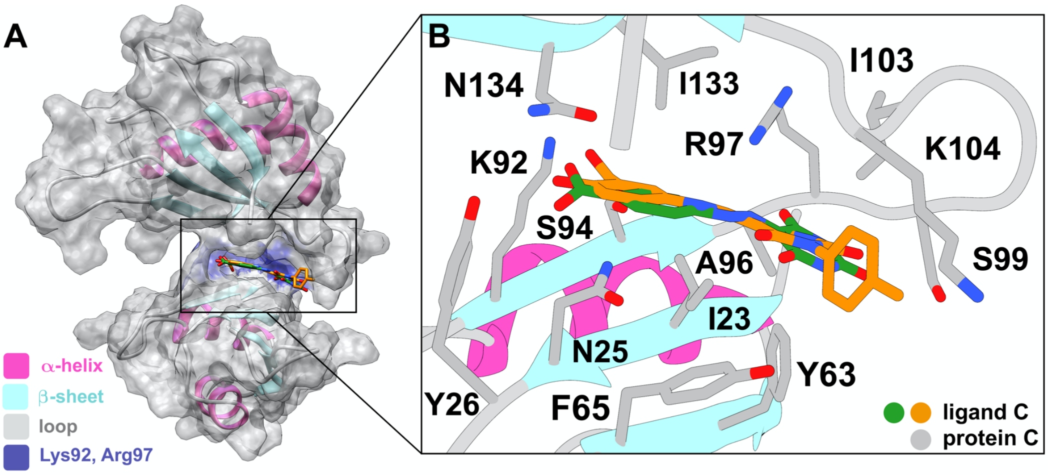

Figure S7. (**A**) Surface representation of HuR RRM1/2 (PDB code 4ED5) with the two docked ligands STK018404 and STL170802. (**B**) Docking pose in licorice fashion of compound STK018404 and STL170802 (with labeled residues within 4 Å of the ligands.

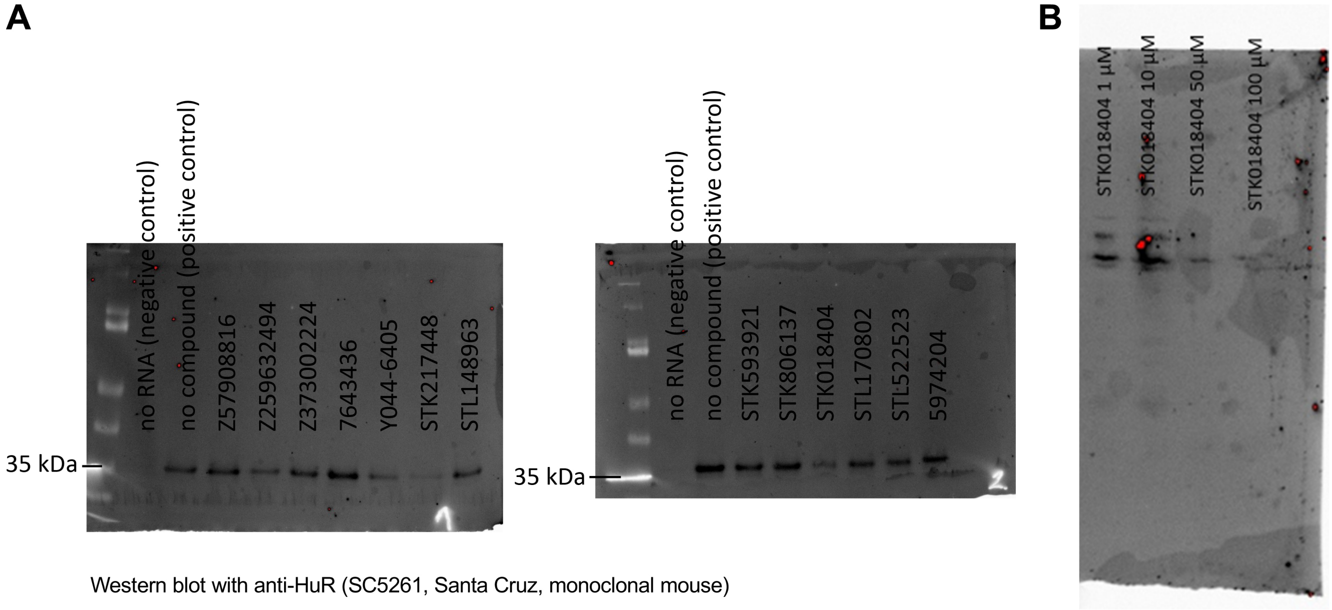

Figure S8. Uncropped western blot gels corresponding to the RNA pull-down assays shown in Figure 4 in the main text. (**A**) RNA–protein pull-down of HuR with its target RNA motif (AUUUUUAUUUU) in the absence (positive control) or presence of the respective compounds (final concentration 100µM). RNA-bound proteins were analyzed on western blots detecting HuR. A negative control that does contain RNA and a positive control with no compound were included. (**B**) RNA–protein pull-down of HuR as described in (A) with different concentrations of the compound STK018404.

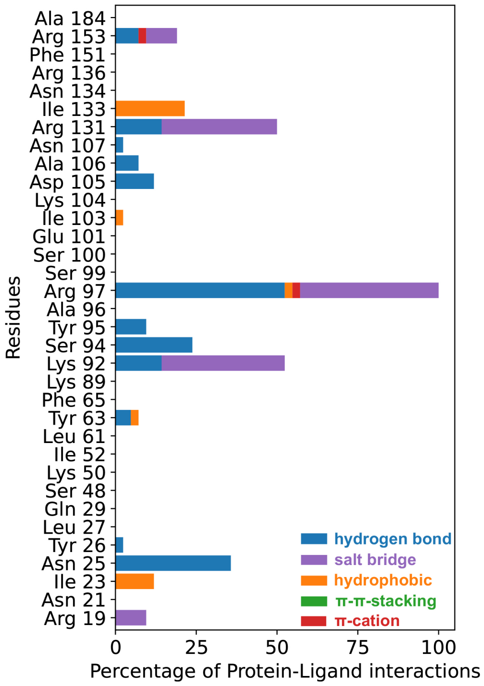

Figure S9. PLIF of the twenty-three poses obtained upon docking of the decoys generated with the DUDE-Z webserver [1].

Table S1. List of previously reported small molecule inhibitors of the HuR protein as of January 2023.

#	Compound name	Status	IC_{50} [μM]	PubChem ID	Ref.
**1**	MS-444	*In vivo*	2.1-40.7	132904	[2]
**2**	Dehydromutactin	*In vitro*	39.0-130.0	54685140	[2]
**3**	Okicenone	*In vitro*	0.53-2.9	132015	[2]
**4**	Quercetin	*In cell*	5.78-31.04	5280343	[3]
**5**	6n	*In cell*	0.015	137652621	[4]
**6**	24	*In vitro*	-	-	[5]
**7**	CMLD-1	*In vitro*	5.98	16060621	[6]
**8**	CMLD-4	*In vitro*	5.22	24868003	[6]
**9**	CMLD-5	*In vitro*	9.13	16746465	[6]
**10**	CMLD-6	*In vitro*	8.88	16746321	[6]
**11**	CMLD-3	*In vitro*	7.11	16746461	[6]
**12**	31	*In vitro*	44.4	265580	[7]
**13**	32	*In vitro*	21.7	137651476	[7]
**14**	23	*In vitro*	41.0	66672	[7]
**15**	30	*In vitro*	41.9	54677920	[7]
**16**	MPT0B098	*In vitro*	0.08-0.51	50898338	[8]
**17**	SP600125	*In vitro*	0.12-16.0	8515	[9]
**18**	Trichostatin A	*In vitro*	-	444732	[9]
**19**	5-aza-2-deoxycitidine	*In vivo*	0.01-0.05	460487	[9]
**20**	N-Benzylcantharidinamide	*In vitro*	50.0-100.0	-	[10]
**21**	Triptolide	*In vivo*	0.01-0.03	107985	[11]
**22**	Leptomycin B	*In vivo*	0.01	6917907	[12]
**23**	Selinexor	*In vivo*	0.12-0.22	71481097	[13]

**24**	Latrunculin A	*In vitro*	0.33-0.76	445420	[14]
**25**	Blebbistatin	*In cell*	0.43-22.8	3476986	[14]
**26**	Mitoxantrone	*In vivo*	0.01-1.13	4212	[5]
**27**	Suramin	*In vivo*	732	5361	[15]
**28**	NSC 5836	*In vitro*	14.7	3034178	[7]
**29**	NSC 7572	*In vitro*	41.0	66672	[7]
**30**	NSC 44750	*In vitro*	26.9	239577	[7]
**31**	NSC 50648	*In vitro*	97.4	242249	[7]
**32**	NSC 62685	*In vitro*	16.7	247668	[7]
**33**	NSC 123418	*In vitro*	69.9	276150	[7]
**34**	NSC 143491	*In vitro*	21.7	9575999	[7]
**35**	NSC 227186	*In vitro*	41.9	54706138	[7]
**36**	NSC 651084	*In vitro*	17.4	6713252	[7]
**37**	Myricetin	*In vitro*	1.0	5281672	[3]
**38**	b-40	*In cell*	0.38	1806245	[16]
**39**	Ellagic acid	*In vitro*	0.6	5281855	[3]
**40**	(-)-Epicatechin gallate	*In vitro*	0.2	107905	[3]
**41**	Rhamnetin	*In vitro*	1.2	5281691	[3]
**42**	Cryptotanshinone	*In cell*	6.0	160254	[17]
**43**	(-)-Epigallocatechin gallate	*In vitro*	0.2	65064	[3]
**44**	Dihydrotanshinone (DHTS)	*In vivo*	0.84-1.2	5316743	[17]
**45**	6a	*In cell*	0.0128	132251303	[4]
**46**	b-41	*In cell*	6.21	58739563	[16]
**47**	b-68	*In cell*	-	1801337	[16]
**48**	a-63	*In cell*	-	6161395	[16]

**49**	Azaphilone-9	*In vitro*	51.9-67.6	57326411	[18]
**50**	CMLD-2	*In vitro*	4.44	16746438	[6]
**51**	Tanshinone I	*In cell*	-	114917	[17]
**52**	HuR inhibitor 5	*In cell*	15-27	3634762	[19]
**53**	1	*In vitro*	-	156010691	[20]
**54**	2	*In vitro*	-	155560371	[20]
**55**	3	*In vitro*	-	155517914	[20]
**56**	4	*In vitro*	-	155540762	[20]
**57**	Hyperoside	*In vitro*	-	5281643	[21]
**58**	Rutin	*In vitro*	-	5280805	[21]
**59**	Isovitexin	*In vitro*	-	162350	[21]
**60**	Vitexin-2-O-rhamnoside	*In vitro*	-	5282151	[21]
**61**	Aesculin	*In vitro*	-	5281417	[21]
**62**	Novobiocin	*In vitro*	-	54675769	[21]
**63**	Chlorogenic acid	*In vitro*	-	1794427	[21]
**64**	7-hydroxymatairesinol	*In vitro*	-	44147426	[21]
**65**	Colchicoside	*In vitro*	-	92763	[21]
**66**	Asiaticoside	*In vitro*	-	11954171	[21]
**67**	Embelin	*In cell*	-	3218	[22]
**68**	KH-3	*In cell*	3.34	45138018	[23]
**69**	KH-3 derivative 1 (1c)	*In cell*	4.74	155343380	[23]
**70**	KH-3 derivative 2 (7c)	*In cell*	1.31	11187381	[23]

Table S2. Docking scores of the thirteen experimentally tested ligands (grey cell) and the known HuR inhibitors.

Compound name	Docking score [kcal/mol]	Compound name	Docking score [kcal/mol]
Z259632494	*-8.646*	NSC 44750	-5.949
Z57908816	*-4.494*	NSC 50648	-5.507
7643436	*-8.618*	NSC 62685	-6.261
STK217448	*-8.287*	NSC 123418	-2.736
STL170802	*-8.623*	NSC 143491	-5.803
5974204	*-8.561*	NSC 227186	-5.485
Y044-6405	*-9.002*	NSC 651084	-4.937
STK018404	*-10.154*	Myricetin	-5.630
Z373002224	*-6.083*	b-40	-5.897
STK806137	*-8.681*	Ellagic acid	-5.427
STK593921	*-5.014*	(-)-Epicatechin gallate	-6.149
STL148963	*-3.350*	Rhamnetin	-2.508
STL522523	*-9.201*	Cryptotanshinone	-3.978
MS-444	-5.226	(-)-Epigallocatechin gallate	-6.212
Dehydromutactin	-5.403	Dihydrotanshinone (DHTS)	-3.963
Okicenone	-5.514	6a	-5.253
Quercetin	-6.475	b-41	-4.969
6n	-4.151	b-68	-5.149
24	-4.590	a-63	-4.498
CMLD-1	-4.586	Azaphilone-9	-3.784
CMLD-4	-3.667	CMLD-2	-3.965
CMLD-5	-3.661	Tanshinone I	-4.690

CMLD-6	-4.045	HuR inhibitor 5	-4.320
CMLD-3	-3.684	1	-3.163
31	-3.209	2	-4.940
32	-5.874	3	-6.945
23	-5.249	4	-5.393
30	-5.132	Hyperoside	-4.485
MPT0B098	0.090	Rutin	-6.708
SP600125	-7.062	Isovitexin	-5.854
Trichostatin A	-3.380	Vitexin-2-O-rhamnoside	-6.320
5-aza-2-deoxycitidine	-4.140	Aesculin	-5.816
N-Benzylcantharidinamide	-4.437	Novobiocin	-5.038
Triptolide	-2.731	Chlorogenic acid	-6.284
Leptomycin B	-5.892	7-hydroxymatairesinol	-6.507
Selinexor	-5.084	Colchicoside	-5.767
Latrunculin A	-4.269	Asiaticoside	-6.402
Blebbistatin	-4.754	Embelin	-2.156
Mitoxantrone	-4.978	KH-3	-3.387
Suramin	-8.556	KH-3 derivative 1 (1c)	-5.417
NSC 5836	-3.913	KH-3 derivative 2 (7c)	-4.980
NSC 7572	-5.249		

Table S3. Predicted off-targets classified by the estimated binding probability (in %) for the 32 representatives of the 70 known HuR known inhibitors (Table S1) and our thirteen experimentally tested molecules, including our identified hit STK18404. Ligands are colored according to the probability range with the largest number of predicted off-targets.

	Target Probability in 100 entries [%]							
**Drug**	0*	1-9	10-15	16-20	21-39	40-50	51-80	81-100
**1**	0	0	100	0	0	0	0	0
**DHTS**	41	0	53	0	0	0	0	6
**6a**	0	0	100	0	0	0	0	0
**b-40**	32	0	68	0	0	0	0	0
**Quercetin**	0	0	0	1	9	6	16	69
**Hyperoside**	78	0	10	1	0	2	1	8
**Chlorogenic acid**	62	0	34	0	2	0	1	1
**Mitoxantrone**	85	0	0	0	0	0	0	15
**CMLD-2**	0	0	99	0	0	0	0	1
**KH-3 d1**	65	0	35	0	0	0	0	0
**NSC 62685**	99	0	1	0	0	0	0	0
**Azaphilone-9**	56	0	44	0	0	0	0	0
**Embelin**	47	0	50	0	3	0	0	0
**MPT0B098**	0	0	100	0	0	0	0	0
**Latrunculin**	100	0	0	0	0	0	0	0
**Leptomycin B**	0	0	100	0	0	0	0	0
**MS-444**	no off-target found#							
**N-Benzylcantharidinamide**	46	0	54	0	0	0	0	0
**Selinexor**	24	0	76	0	0	0	0	0
**SP600125**	57	43	0	0	0	0	0	0
**Suramin**	100	0	0	0	0	0	0	0
**Dehydromutactin**	78	0	22	0	0	0	0	0
**Okicenone**	no off-target found#							

**23**	32		68	0	0	0	0	0
**30**	100		0	0	0	0	0	0
**31**	100		0	0	0	0	0	0
**32**	81		19	0	0	0	0	0
**Trichostatin A**	0	0	100	0	0	0	0	0
**5-aza-2-deoxycitidine**	94	6	0	0	0	0	0	0
**Triptolide**	no off-target found#							
**Blebistatin**	3		97	0	0	0	0	0
**HuR inhibitor 5**	0		100	0	0	0	0	0
**5974204**	31		69	0	0	0	0	0
**7643436**	0		100	0	0	0	0	0
**STK018404**	93		7	0	0	0	0	0
**STL170802**	100		0	0	0	0	0	0
**STK217448**	0	0	100	0	0	0	0	0
**STK593921**	0	0	100	0	0	0	0	0
**STK806137**	0	0	100	0	0	0	0	0
**STL148963**	62	0	38	0	0	0	0	0
**STL522523**	8	0	92	0	0	0	0	0
**Y044-6405**	65	1	34	0	0	0	0	0
**Z57908816**	0	0	99	0	0	1	0	0
**Z373002224**	88	0	12	0	0	0	0	0
**Z259632494**	no off-target found#							

* Probability equal to 0% means that the confidence of the off-target prediction is very low, due to limited similarity between the query ligand and the known actives present in the SwissTargetPrediction database. In this regard, the webserver combines 2D and 3D similarity measures between the query and the database molecules, with different thresholds [24]. This might result in predicted off-targets with a certain number of know actives similar in 3D to the query ligand, but no known active similar in 2D, in which case the probability of the investigated but no known active similar in 2D, in which case the probability of the investigated
# "No off-target found" indicates that there are no active molecules in the SwissTargetPrediction database similar to the query molecule and thus no off-target was predicted.

Table S4. Protein class of the predicted top off-targets for the 32 representatives of the known HuR known inhibitors and the thirteen experimentally tested ligands. Color code for the ligands is the same as in Table S3.

Drug	Top Targets			
**1**	Kinase	Family A GPCR		
**DHTS**	Aldose reductase	Acetylcholinesterase		
**6a**	Kinase	Family A GPCR		
**b-40**	Kinase	Family A GPCR		
**Quercetin**	Kinase	Lyase	Protease	
**Hyperoside**	Aldose reductase	Carbonic anhydrases 2, 4, 7, 12	NADPH oxidase 4	Adrenergic receptor alpha-2
**Chlorogenic acid**	Aldose reductase	Aldo-keto reductase family 1 member B10		
**Mitoxantrone**	Electrochemical transporter	Kinase	Family A GPCR	
**CMLD-2**	Eukaryotic translation inhibition factor 4H	Family A GPCR	Kinase	Protease
**KH-3 d1**	Eraser	Protease		
**NSC 62685**	5′-nucleotidase			
**Azaphilone-9**	Kinase	Family A GPCR		
**Embelin**	Histone Acetyltransferase	Macrophage-expressed gene 1 protein	Arachidonate 5-lipoxygenase	
**MPT0B098**	Family A GPCR	Kinase		
**Latrunculin**				
**Leptomycin B**	Family A, B, C GPCR	Eraser	Protease	
**MS-444**	no off-target found			
**N-Benzylcantharidinamide**	Family A GPCR	Protease	Reader	
**Selinexor**	Kinase	Protease	Family A GPCR	
**SP600125**	Kinase	Family A GPCR		
**Suramin**				
**Dehydromutactin**	Oxidoreductase	Voltage-gated ion channel		
**Okicenone**	no off-target found			
**23**	Kinase	Lyase	Family A GPCR	

**30**	Kinase	Protease		
**31**				
**32**	Protease	Family A GPCR		
**Trichostatin A**	Eraser	Protease	Family A GPCR	
**5-aza-2-deoxycitidine**	Protease	Hydrolase		
**Triptolide**	no off-target found			
**Blebistatin**	Kinase	Protease	Family A GPCR	
**HuR inhibitor 5**	Kinase	Protease	Family A GPCR	
**5974204**	Kinase	Family A GPCR		
**7643436**	Kinase	Lyase		
**STK018404**	Oxidoreductase	Transferase	Kinase	
**STL170802**	Eraser	Phosphodiesterase	Lyase	
**STK217448**	Family A GPCR	Kinase	Voltage-gated ion channel	
**STK593921**	Kinase	Potease	Eraser	Family A GPCR
**STK806137**	Protease	Lyase	Kinase	
**STL148963**	Protease	Family A GPCR	Fatty acid protein binding family	Family A GPCR
**STL522523**	Protease	Kinase		
**Y044-6405**	Eraser	Kinase	Lyase	
**Z57908816**	Adenosine alpha 2a receptor			
**Z373002224**	Kinase	Family A GPCR		
**Z259632494**	no off-target found			

Table S5. Docking scores of the twenty-three successfully docked decoys.

Compound name	Docking score [kcal/mol]	Compound name	Docking score [kcal/mol]
ZINC000008220417	-6.929	ZINC000005112560	-5.484
ZINC000005340116	-6.782	ZINC000712777334	-5.352
ZINC000006827695	-6.687	ZINC000008214677	-5.351
ZINC000000895180	-6.388	ZINC000005828408	-5.340
ZINC000002044203	-5.933	ZINC000013542400	-5.269
ZINC000001542148	-5.930	ZINC000089222544	-5.150
ZINC000004096278	-5.929	ZINC000206744254	-5.082
ZINC000004096089	-5.866	ZINC000136274150	-5.044
ZINC000001529331	-5.856	ZINC000003869232	-5.033
ZINC000020157024	-5.795	ZINC000005840872	-4.999
ZINC000029391179	-5.487	ZINC000006090886	-4.492
ZINC000001850486	-5.484		

References

1. Stein, R. M.; Yang, Y.; Balius, T. E.; O'Meara, M. J.; Lyu, J.; Young, J.; Tang, K.; Shoichet, B. K.; Irwin, J. J. Property-Unmatched Decoys in Docking Benchmarks. *J. Chem. Inf. Model*. **2021**, 61, 699–714. https://doi.org/10.1021/acs.jcim.0c00598.
2. Meisner, N.-C.; Hintersteiner, M.; Mueller, K.; Bauer, R.; Seifert, J.-M.; Naegeli, H.-U.; Ottl, J.; Oberer, L.; Guenat, C.; Moss, S.; Harrer, N.; Woisetschlaeger, M.; Buehler, C.; Uhl, V.; Auer, M. Identification and Mechanistic Characterization of Low-Molecular-Weight Inhibitors for HuR. Nat. Chem. Biol. **2007**, 3, 508–515. https://doi.org/10.1038/nchembio.2007.14.
3. Kwak, H.-J.; Jeong, K.-C.; Chae, M.-J.; Kim, S.-Y.; Park, W.-Y. Flavonoids Inhibit the AU-Rich Element Binding of HuC. *BMB Rep*. **2009**, 42, 41–46. https://doi.org/10.5483/BMBRep.2009.42.1.041.
4. Manzoni, L.; Zucal, C.; Maio, D. D.; D'Agostino, V. G.; Thongon, N.; Bonomo, I.; Lal, P.; Miceli, M.; Baj, V.; Brambilla, M.; Cerofolini, L.; Elezgarai, S.; Biasini, E.; Luchinat, C.; Novellino, E.; Fragai, M.; Marinelli, L.; Provenzani, A.; Seneci, P. Interfering with HuR–RNA Interaction: Design, Synthesis and Biological Characterization of Tanshinone Mimics as Novel, Effective HuR Inhibitors. *J. Med. Chem*. **2018**, 61, 1483–1498. https://doi.org/10.1021/acs.jmedchem.7b01176.
5. D'Agostino, V. G.; Adami, V.; Provenzani, A. A Novel High Throughput Biochemical Assay to Evaluate the HuR Protein-RNA Complex Formation. *PLoS ONE* **2013**, 8, e72426. https://doi.org/10.1371/journal.pone.0072426.
6. Wu, X.; Lan, L.; Wilson, D. M.; Marquez, R. T.; Tsao, W.; Gao, P.; Roy, A.; Turner, B. A.; McDonald, P.; Tunge, J. A.; Rogers, S. A.; Dixon, D. A.; Aubé, J.; Xu, L. Identification and Validation of Novel Small Molecule Disruptors of HuR-MRNA Interaction. *ACS Chem. Biol*. **2015**, 10, 1476–1484. https://doi.org/10.1021/cb500851u.
7. Wang, Z.; Bhattacharya, A.; Ivanov, D. N. Identification of Small-Molecule Inhibitors of the HuR/RNA Interaction Using a Fluorescence Polarization Screening Assay Followed by NMR Validation. *PLOS ONE* **2015**, 10, e0138780. https://doi.org/10.1371/journal.pone.0138780.
8. Cheng, Y.-C.; Liou, J.-P.; Kuo, C.-C.; Lai, W.-Y.; Shih, K.-H.; Chang, C.-Y.; Pan, W.-Y.; Tseng, J. T.; Chang, J.-Y. MPT0B098, a Novel Microtubule Inhibitor That Destabilizes the Hypoxia-Inducible Factor-1α MRNA through Decreasing Nuclear–Cytoplasmic Translocation of RNA-Binding Protein HuR. *Mol. Cancer Ther*. **2013**, 12, 1202–1212. https://doi.org/10.1158/1535-7163.MCT-12-0778.
9. Hostetter, C.; Licata, L. A.; Costantino, C. L.; Witkiewicz, A.; Yeo, C.; Brody, J. R.; Keen, J. C. Cytoplasmic Accumulation of the RNA Binding Protein HuR Is Central to Tamoxifen Resistance in Estrogen Receptor Positive Breast Cancer Cells. *Cancer Biol. Ther*. **2008**, 7, 1496–1506. https://doi.org/10.4161/cbt.7.9.6490.
10. Lee, J.-Y.; Chung, T.-W.; Choi, H.-J.; Lee, C. H.; Eun, J. S.; Han, Y. T.; Choi, J.-Y.; Kim, S.-Y.; Han, C.-W.; Jeong, H.-S.; Ha, K.-T. A Novel Cantharidin Analog N-Benzylcantharidinamide Reduces the Expression of MMP-9 and Invasive Potentials of Hep3B via Inhibiting Cytosolic Translocation of HuR. *Biochem. Biophys. Res. Commun*. **2014**, 447, 371–377. https://doi.org/10.1016/j.bbrc.2014.04.035.
11. Sun, L.; Zhang, S.; Jiang, Z.; Huang, X.; Wang, T.; Huang, X.; Li, H.; Zhang, L. Triptolide Inhibits COX-2 Expression by Regulating MRNA Stability in TNF-α-Treated A549 Cells. *Biochem. Biophys. Res. Commun*. **2011**, 416, 99–105. https://doi.org/10.1016/j.bbrc.2011.11.004.
12. Mutka, S. C.; Yang, W. Q.; Dong, S. D.; Ward, S. L.; Craig, D. A.; Timmermans, P. B. M. W. M.; Murli, S. Identification of Nuclear Export Inhibitors with Potent Anticancer Activity In Vivo. *Cancer Res*. **2009**, 69, 510–517. https://doi.org/10.1158/0008-5472.CAN-08-0858.
13. Hing, Z. A.; Mantel, R.; Beckwith, K. A.; Guinn, D.; Williams, E.; Smith, L. L.; Williams, K.; Johnson, A. J.; Lehman, A. M.; Byrd, J. C.; Woyach, J. A.; Lapalombella, R. Selinexor Is Effective in Acquired Resistance to Ibrutinib and Synergizes with Ibrutinib in Chronic Lymphocytic Leukemia. *Blood* **2015**, 125, 3128–3132. https://doi.org/10.1182/blood-2015-01-621391.
14. Doller, A.; Badawi, A.; Schmid, T.; Brauß, T.; Pleli, T.; Zu Heringdorf, D. M.; Piiper, A.; Pfeilschifter, J.; Eberhardt, W. The Cytoskeletal Inhibitors Latrunculin A and Blebbistatin Exert Antitumorigenic Properties in Human Hepatocellular Carcinoma Cells by Interfering with Intracellular HuR Trafficking. *Exp. Cell Res*. **2015**, 330, 66–80. https://doi.org/10.1016/j.yexcr.2014.09.010.
15. Kakuguchi, W.; Nomura, T.; Kitamura, T.; Otsuguro, S.; Matsushita, K.; Sakaitani, M.; Maenaka, K.; Tei, K. Suramin, Screened from an Approved Drug Library, Inhibits HuR Functions and

Attenuates Malignant Phenotype of Oral Cancer Cells. *Cancer Med*. **2018**, 7, 6269–6280. https://doi.org/10.1002/cam4.1877.

16. Chae, M.-J.; Sung, H. Y.; Kim, E.-H.; Lee, M.; Kwak, H.; Chae, C. H.; Kim, S.; Park, W.-Y. Chemical Inhibitors Destabilize HuR Binding to the AU-Rich Element of TNF-α MRNA. *Exp. Mol. Med*. **2009**, 41, 824. https://doi.org/10.3858/emm.2009.41.11.088.
17. D'Agostino, V. G.; Lal, P.; Mantelli, B.; Tiedje, C.; Zucal, C.; Thongon, N.; Gaestel, M.; Latorre, E.; Marinelli, L.; Seneci, P.; Amadio, M.; Provenzani, A. Dihydrotanshinone-I Interferes with the RNA-Binding Activity of HuR Affecting Its Post-Transcriptional Function. *Sci. Rep*. **2015**, 5, 16478. https://doi.org/10.1038/srep16478.
18. Kaur, K.; Wu, X.; Fields, J. K.; Johnson, D. K.; Lan, L.; Pratt, M.; Somoza, A. D.; Wang, C. C. C.; Karanicolas, J.; Oakley, B. R.; Xu, L.; De Guzman, R. N. The Fungal Natural Product Azaphilone-9 Binds to HuR and Inhibits HuR-RNA Interaction in Vitro. *PLOS ONE* **2017**, 12, e0175471. https://doi.org/10.1371/journal.pone.0175471.
19. Filippova, N.; Yang, X.; Ananthan, S.; Sorochinsky, A.; Hackney, J. R.; Gentry, Z.; Bae, S.; King, P.; Nabors, L. B. Hu Antigen R (HuR) Multimerization Contributes to Glioma Disease Progression. *J. Biol. Chem*. **2017**, 292, 16999–17010. https://doi.org/10.1074/jbc.M117.797878.
20. Della Volpe, S.; Nasti, R.; Queirolo, M.; Unver, M. Y.; Jumde, V. K.; Dömling, A.; Vasile, F.; Potenza, D.; Ambrosio, F. A.; Costa, G.; Alcaro, S.; Zucal, C.; Provenzani, A.; Di Giacomo, M.; Rossi, D.; Hirsch, A. K. H.; Collina, S. Novel Compounds Targeting the RNA-Binding Protein HuR. Structure-Based Design, Synthesis, and Interaction Studies. *ACS Med. Chem. Lett*. **2019**, 10, 615–620. https://doi.org/10.1021/acsmedchemlett.8b00600.
21. Vasile, F.; Della Volpe, S.; Ambrosio, F. A.; Costa, G.; Unver, M. Y.; Zucal, C.; Rossi, D.; Martino, E.; Provenzani, A.; Hirsch, A. K. H.; Alcaro, S.; Potenza, D.; Collina, S. Exploration of Ligand Binding Modes towards the Identification of Compounds Targeting HuR: A Combined STD-NMR and Molecular Modelling Approach. *Sci. Rep*. **2018**, 8, 13780. https://doi.org/10.1038/s41598-018-32084-z.
22. Dai, Y.; Jiao, H.; Teng, G.; Wang, W.; Zhang, R.; Wang, Y.; Hebbard, L.; George, J.; Qiao, L. Embelin Reduces Colitis-Associated Tumorigenesis through Limiting IL-6/STAT3 Signaling. *Mol. Cancer Ther*. **2014**, 13, 1206–1216. https://doi.org/10.1158/1535-7163.MCT-13-0378.
23. Wu, X.; Ramesh, R.; Wang, J.; Zheng, Y.; Armaly, A. M.; Wei, L.; Xing, M.; Roy, S.; Lan, L.; Gao, F. P.; Miao, Y.; Xu, L.; Aubé, J. Small Molecules Targeting the RNA-Binding Protein HuR Inhibit Tumor Growth in Xenografts. *J. Med. Chem*. **2023**, 66, 2032–2053. https://doi.org/10.1021/acs.jmedchem.2c01723.
24. Davies, M.; Nowotka, M.; Papadatos, G.; Dedman, N.; Gaulton, A.; Atkinson, F.; Bellis, L.; Overington, J. P. ChEMBL Web Services: Streamlining Access to Drug Discovery Data and Utilities. *Nucleic Acids Res*. **2015**, 43, W612–W620. https://doi.org/10.1093/nar/gkv352.
